# Matrine induces senescence of human glioblastoma cells through suppression of the IGF1/PI3K/AKT/p27 signaling pathway

**DOI:** 10.1002/cam4.1720

**Published:** 2018-08-05

**Authors:** Wenjing Zhou, Jiwei Wang, Qichao Qi, Zichao Feng, Bin Huang, Anjing Chen, Di Zhang, Wenjie Li, Qing Zhang, Rolf Bjerkvig, Xingang Li, Jian Wang

**Affiliations:** ^1^ Department of Neurosurgery Qilu Hospital of Shandong University Brain Science Research Institute Key Laboratory of Brain Functional Remodeling Shandong University Jinan Shandong China; ^2^ Department of Biomedicine K G Jebsen Brain Tumor Research Center University of Bergen Bergen Norway

**Keywords:** glioblastoma, IGF1/PI3K/p27 signaling pathway, matrine, senescence

## Abstract

**Background:**

Matrine, a traditional Chinese medicine, has recently been shown to have antitumor properties in diverse cancer cells. Here, we explored the effect of matrine on human glioblastoma multiforme (GBM) cells.

**Methods:**

Glioblastoma multiforme cell lines were treated with matrine to assess proliferation and viability using EdU and CCK8 assays. SA‐β‐gal assays were used to evaluate cellular senescence, and a cytokine array and ELISA assay were used to screen for secreted cytokines altered in GBM cells after matrine treatment. Immunohistochemistry and Western blot analysis were performed to evaluate protein levels in matrine‐treated cell lines and in samples obtained from orthotopic xenografts. Specific activators of AKT and IGF1 were used to identify the pathways mediating the effect.

**Results:**

Matrine potently inhibited growth of GBM cell lines in vitro. Based on in situ assays, growth arrest induced by matrine was primarily achieved through induction of cellular senescence. Matrine treatment led to decreased expression of proteins involved in promoting cell growth, IGF1, PI3K, and pAKT. Exposure of cells to a small molecule activating AKT (SC79) and recombinant IGF1 led to a reduced number of senescent SA‐β‐gal‐positive cells in the presence of matrine. Finally, matrine inhibited growth of orthotopic xenografts established from luciferase‐stable‐U251 or luciferase‐stable‐P3 cells and prolonged overall survival in mice.

**Conclusions:**

These results indicated that matrine arrested cell growth through inhibition of IGF1/PI3K/AKT signaling. Matrine warrants further investigation as a potential therapy in the treatment of patients with GBM.

## 1 INTRODUCTION

Glioblastoma multiforme (GBM; WHO grade IV) is the most common malignant brain tumor, with characteristics of rapid progression, poor curative effect, and unfavorable prognosis.^1^, ^2^ Despite advances in combination treatments consisting of radiotherapy and chemotherapy, such as temozolomide which is considered the first‐line adjuvant treatment for all patients,^3^, ^4^, ^5^ the 5‐year survival rate of GBM patients remains dismally at less than 5%.^6^ Therefore, more effective therapies for the treatment of GBM are desperately needed.

Studies in the past decade have greatly advanced our understanding of the genetic alterations that underlie the pathogenesis of glioblastoma. Such genetic information provides investigators with a basic map of proteins and/or pathways that might be specifically targeted with molecular compounds and thereby enhance efficacy of cancer treatment. An important resource for candidate molecules in the modern‐day treatment of human cancer is traditional Chinese medicine. Many of these medicines have been in clinical use for centuries for a broad spectrum of human conditions, and, yet, we have a poor understanding of how and why they work. In today's research environment, we finally have an opportunity to realize the full potential of these medicines, but only if we have knowledge of the molecular pathways they regulate.

Matrine, an alkaloid extracted from sophora flavescens, is one such traditional Chinese medicine with a history of clinical application of more than 2000 years.^7^ It has long been used for the treatment of viral hepatitis, cardiac arrhythmia, and inflammations of the skin.^8^ Recent results have demonstrated that matrine possesses antitumor activities against several types of cancer cells.^9^, ^10^ In this study, we examined the effect of matrine on GBM cells in vitro and in vivo. We demonstrate that matrine exerts a potent antitumor effect on GBM cells primarily through the induction of cellular senescence and inhibition of one of the main pathways corrupted in GBM, PI3K/AKT.^11^, ^12^, ^13^, ^14^ These results indicate that matrine has promise as a chemotherapeutic agent in the treatment of GBM patients.

## 2 MATERIALS AND METHODS

### 2.1 Ethics statement

Mice were housed in the SPF animal facility of Qilu Hospital of Shandong University. All animal procedures were approved by the Medical Ethics Committee of Shandong University and performed in accordance with the guidelines of the Institutional Animal Care and Use Committee of Shandong University (Jinan, Shandong, China).

### 2.2 Cell lines and cultures

Normal human astrocytes (NHA) were purchased from BeNa Culture Collection (BNCC341796, Beijing, China), and human glioma cell lines (U251, TCHu 58, and U87 MG, TCHu 138) were obtained from the Cell Bank of Type Culture Collection of Chinese Academy of Sciences (Shanghai, China). P3, a primary GBM cell line propagated in vivo, GFP‐luciferase‐stable U251, LN18, and LN229 were kindly provided by Prof. Rolf Bjerkvig, University of Bergen (Bergen, Norway). All the cell lines have been authenticated through DNA fingerprinting and cross‐species checks. All cells were cultured in Dulbecco's modified Eagle's medium (DMEM, H30022.01B, Thermo Fisher Scientific; Waltham, MA, USA) with 10% fetal bovine serum (FBS, 10082147 Hyclone; GE Healthcare Life Sciences; Pittsburgh, PA, USA) at 37°C in a 5% CO_2_‐humidified atmosphere. Cells were treated with matrine (M5319‐500MG, Sigma‐Aldrich, St. Louis, MO, USA), an activator of pAKT SC79 (305834‐79‐1; Sigma‐Aldrich), or an inhibitor of PI3K LY294002 (934389‐88‐5; Sigma‐Aldrich) at the concentrations indicated in the text. Dimethyl sulfoxide (DMSO, W387520, Sigma‐Aldrich) was used as the vehicle control.

### 2.3 Cell proliferation and TUNEL assays

GBM cells (10^4^ cells/well) were plated in 96‐well plates, and cell proliferation was evaluated at specific time points using the CCK‐8 kit (CCK‐8; CK04‐500, Dojindo Laboratories; Kumamoto, Japan) according to the manufacturer's instructions. For the TUNEL assay, cells were seeded onto glass slides in 24‐well culture plates (10^5^ cells/well) for 24 hours and subsequently treated with drugs for an additional 48 hours in serum‐free DMEM. Cells were stained using the TUNEL assay with the TACS_2 TdT‐Fluor in situ cell death detection kit (22849, Roche; Indianapolis, IN, USA) according to the manufacturer's instructions. Slides were evaluated under fluorescence microscopy, and images were scanned with a DP71 CCD digital camera. All experiments were repeated three times.

### 2.4 Flow cytometry for cell cycle analysis and apoptosis

For cell cycle analysis, cells were harvested using 0.25% trypsin‐109 EDTA (ROO1100, Thermo Fisher Scientific; Waltham, MA, USA), centrifuged (300 g), and rinsed once with cold PBS. The pellet was resuspended in ice‐cold 70% ethanol and stored at −20°C. Cells were subsequently harvested, incubated with propidium iodide (50 μg/mL, 550825, BD Biosciences; San Diego, CA, USA) and RNase A (100 μg/mL, 19101, Qiagen, Hilden, Germany) for 30 minutes, and analyzed using flow cytometry (BD Accuri^™^ C6 PLUS, BD Biosciences).

For detection of apoptosis, both adherent and floating cells were harvested, resuspended in binding buffer, rinsed twice in PBS, and incubated with propidium iodide (5 μL) and annexin V‐FITC (5 μL) in Annexin V Binding Buffer (100 μL, 556547, FITC Annexin V Apoptosis Detection Kit I, BD Biosciences, NJ, USA) according to the manufacturer's instructions. Apoptotic cells were detected using flow cytometry (ACEA Biosciences; San Diego, CA, USA), and the results were analyzed using the software Flowjo (Tree Star; Ashland, OR, USA). All experiments were repeated three times.

### 2.5 Senescence‐associated‐β‐galactosidase (SA‐β‐gal) assay

The Senescence‐Associated‐β‐Galactosidase Staining Kit (9860, Cell Signaling Technology; Danvers, MA, USA) was used to stain senescent cells. Control cells seeded into 6‐well plates at 100 000 cells/well were confluent after 2 days. Cells were split in order to avoid false‐positive staining due to confluent growth of cells, and the overall observation time for the experiments was extended. Briefly, cells in culture were fixed with 1X fixative solution for 15 minutes at room temperature and incubated with X‐gal staining solution overnight at 37°C in an incubator without CO_2_ according to the manufacturer's protocol. SA‐β‐gal‐positive cells were examined under bright‐field microscopy and were quantified using the software ImageJ (National Institutes of Health, Bethesda, MA, USA) cell counter taken for three random fields per sample (n = 3 samples).

### 2.6 Immunofluorescence staining

Cells in culture were rinsed with PBS, fixed in 4% paraformaldehyde in PBS for 15 minutes, permeabilized with 0.5% Triton X‐100 in PBS for 10 minutes, blocked in 3% BSA for 30 minutes, and incubated with rabbit antiphospho‐histone H2AX (γH2AX; Ser139) antibody (#2577S, Cell Signaling Technology) at a dilution of 1:200 in 5% bovine serum albumin in PBS overnight at 4°C. Cells were rinsed with PBS and incubated with fluorescently tagged secondary antibodies, Alexa Fluor 647‐conjugated anti‐rabbit IgG (#A27040, Thermo Fisher Scientific) at a dilution of 1:200 in PBS for 1 hour. Nuclei were stained with DAPI (1 μg/mL in PBS) for 5 minutes in the dark. Slides were examined under fluorescence microscopy, and images were acquired using a DP71 CCD (charge‐coupled device) digital camera (Olympus; Waltham, MA, USA). The percentage of γH2AX + nuclei‐positive cell was determined using images taken for three random fields per sample (n = 3 samples).

### 2.7 Immunohistochemistry (IHC)

Tumor samples were fixed in 4% formaldehyde overnight at 4°C, paraffin embedded, sectioned (5 μm), and mounted on microscopic slides. Heat‐induced epitope retrieval was performed with a microwave in 1 mmol/L citric acid buffer, pH 7.2, and samples were blocked in 200 μL of blocking buffer (950 μL of TBST/5% BSA + 50 μL of serum from the species of the secondary antibody) for 30 minutes at room temperature and incubated with primary antibody at 4°C overnight. Sections were rinsed with PBS, and detection was performed through standard procedures using horse‐radish peroxidase‐linked secondary antibody (goat anti‐rabbit or anti‐mouse) and diaminobenzidine as substrate (CTS002‐NOV, HRP‐DAB IHC Detection kit, Novus Biologicals, Littleton, CO, USA). Slides were counterstained with Mayer's hematoxylin and evaluated under light microscopy (MC21‐N, Sony ics412 CCD). Primary antibodies used were the following: Ki67, 1:300 (#9449); p27,1:400 (#83630); and γH2AX, 1:400 (#9718S; Cell Signalling Technology). All experiments were repeated three times.

### 2.8 Western blot

Western blot analysis was used to detect the levels of target proteins in GBM cells. Total cell protein extracts were obtained with lysis in radioimmunoprecipitation assay buffer (RIPA; P0013C, Beyotime; Haimen, China) supplemented with a protein inhibitor cocktail (20‐201, Millipore Sigma; Burlington, MA, USA) for 30 minutes. Protein concentrations were evaluated with the BCA assay according to the manufacturer's instructions (Beyotime). Protein lysates (20 μg) were separated using 10% SDS‐polyacrylamide gel electrophoresis (150 V, 60‐90 minutes) and transblotted to polyvinylidene difluoride (PVDF) membranes (GVW2932A, 0.22 μm, Millipore Sigma) using wet transfer (220 mA, 100 minutes). Membranes were then blocked in 5% skim milk in Tris‐buffered saline containing 0.1% Tween‐20 for 1 hour, and incubated with primary antibodies overnight at 4°C. After rinsing, blots were probed with the appropriate secondary antibodies(dilution 1:3000; goat anti‐rabbit: A0208, goat anti‐mouse: A0216, Beyotime). Bands were visualized using the Chemiluminescent HRP Reagents Kit (WBKLS0500, Millipore Sigma). Chemiluminescent signals were imaged using ChemiDoc XRS+ (Bio‐Rad; Hercules, CA, USA) and quantified with ImageJ software. Primary antibodies used were the following: AKT (dilution 1:1000, 10176‐2‐AP; Proteintech, Chicago, IL, USA), pAKT (dilution 1:1000, Ser473; #4060), γH2AX (dilution 1:1000, #9234), p27 (dilution 1:1000, #3686), p21 (dilution 1:1000, #2947), PI3K (dilution 1:500,#4229), p53 (dilution 1:1000, #2523), caspase 3 (dilution 1:1000, #9662S), cleaved caspase 3 (dilution 1:1000, #9661S), PARP (dilution 1:1000, #9532S), cleaved PARP (dilution 1:1000, #5625S), β‐tublin (dilution 1:1000, #2148S), CDK4 (dilution 1:1000, #12790), CDK6 (dilution 1:1000, #13331; Cell Signaling Technology), and Rb (dilution 1:1000, ab181616, Abcam; Cambridge, United Kingdom). GAPDH protein levels were used as a protein loading control (#TA‐09, ZSGB‐BIO; Beijing, China). All experiments were repeated three times.

### 2.9 SiRNA transfections

SiRNAs, negative control and targeting p27 (si‐p27, #12324, Cell Signaling Technology), were transfected into U251 and U87 cells according to the manufacturer's protocol. Transfections were performed with Lipofectamine 2000 (11668027, Thermo Fisher Scientific) in the absence of serum. p27 protein levels in transfected cells were determined by Western blot 48 hours later, and cell senescence was detected using SA β‐gal activity 72 hours later as described above. Three independent experiments were performed.

### 2.10 Cytokine array and Enzyme‐linked Immunosorbent Assay (ELISA)

A RayBio human cytokine array C5 (RayBiotech; Norcross, GA, USA) was used to screen for the secretion of 80 different cytokines in the supernatant of control‐ or matrine‐treated U251 cells in vitro following the manufacturer's protocol. Quantitative ELISAs for IGF1 (R&D Systems, Minneapolis, MN, USA) were performed to verify and quantify cytokine secretion according to the manufacturer's instructions. Absorbance was measured using a microplate reader. Three independent experiments were performed.

### 2.11 Xenografts

GFP‐luciferase‐stable U251 and P3 glioma cells (1 × 10^6^) in total volume of 10 μL of serum‐free DMEM were implanted into the right striatum of athymic mice (male; 4 weeks old; 20‐30 g; Shanghai SLAC Laboratory Animal Co., Ltd; Shanghai, China) at a depth of 2.5 mm using a Hamilton syringe. Implanted mice were randomly divided into three treatment groups: control (PBS), n* *=* *5; low matrine (40 mg/kg/d), n* *=* *5; and high matrine (80 mg/kg/d), n* *=* *5. All animals were i.p. injected every other day starting on day 3 following implantation. Mice were then monitored by bioluminescence imaging every week. Briefly, mice were injected with luciferin (100 mg; Caliper Life Sciences; Waltham, MA, USA), simultaneously anesthetized with isoflurane, and imaged with a cooled charge‐coupled device camera (IVIS‐200, Xenogen; Alameda, CA, USA). Bioluminescence values of tumors were quantitated using the Living Image 2.5 software package (Xenogen). For survival curve, athymic mice (n = 30) were divided into three groups (control, low matrine, and high matrine; 10 mice per group) and the survival periods of mice were recorded. Mice were euthanized after 30 days and perfused with 4% paraformaldehyde in PBS. Brains were fixed with 4% paraformaldehyde in PBS at 4***°***C overnight, dehydrated in 20% sucrose until the tissue sank, embedded in OCT (Thermo Fisher Scientific), and sectioned for hematoxylin and eosin (H&E) staining and immunohistochemistry.

### 2.12 Statistical analysis

Statistical analyses were performed using log‐rank test of Kaplan‐Meier analysis and analysis of variance in randomized blocks in GraphPad Prism 6 software (San Diego, CA, USA) and permutation test implemented with R.^15^ Three independent experiments were performed, and results were expressed as the mean ± the standard deviation (SD). *P*‐values determined from different comparisons are indicated.

## 3 RESULTS

### 3.1 Matrine inhibits proliferation of GBM cells

**Figure 1:**
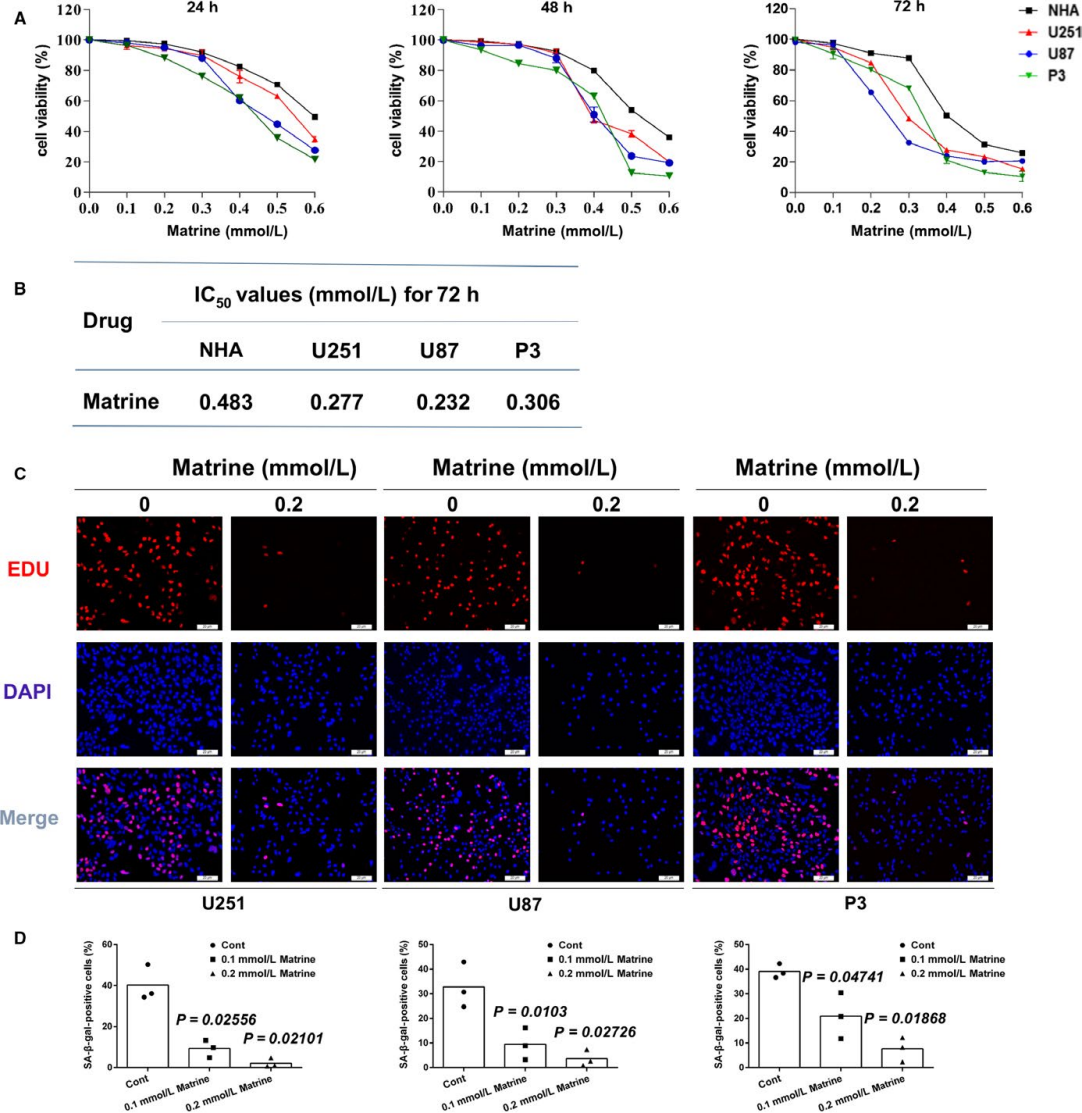
Matrine inhibits proliferation and induces cell cycle arrest in GBM cells. A, Growth curves generated with cell viability results determined from the CCK‐8 assay for normal human astrocytes (NHA), U251, U87, and P3 cells treated with different concentrations of matrine for 24, 48, and 72 h. Data points represent the percentage (%; OD450 untreated/OD450 treated) relative to untreated cells at that time point. B, IC50 values for matrine in three glioma cell lines and NHA. C, U251, U87, and P3 cells were treated with 0.2 mmol/L matrine for 72 h. The cells were stained with Apollo 647 (red, representative of EdU) and the nuclear‐specific dye DAPI (blue). Scale bars = 20 μm. D, Graphic representation of the percentage of EdU‐positive U251, U87, and P3 cells treated with different concentrations of matrine for 72 h. Statistical analyses were performed using permutation test. *P*‐value came from the comparison between treated group and control group. All data are representative of three independent experiments

To begin to determine whether matrine might be an effective chemotherapeutic agent against GBM, we evaluated response to the alkaloid in several GBM cell lines in culture, including U251, U87, and P3, and a normal cell type, NHA. Dose‐response curves based on cell viability data collected from the CCK‐8 assay at 24, 48, and 72 hours indicated that treatment with increasing concentrations of matrine resulted in growth inhibition of all cells tested (Figure 1A). Furthermore, IC_50_ values at 72 hours for glioma cell lines were ~1.5 to 2× less than in NHA indicating that tumor cells were more sensitive to the alkaloid than NHA (Figure 1B).

Proliferation of cell types was also correspondingly decreased under treatment with matrine. Quantification of EdU incorporation revealed a statistically significant decrease in proliferation for both U251, U87, and P3 cell lines at 72 hours after exposure to matrine at 0.1 and 0.2 mmol/L (U251: ~ 40% vs ~ 10% and ~ 3%, U87: ~ 34% vs ~ 8% and ~ 2%, and P3: ~ 39% vs ~ 20% and ~ 5%, control vs 0.1 and 0.2 mmol/L matrine) in Figure 1C,D. EDU and apoptosis assays were also performed on NHA cells treated with matrine at 0.1 and 0.2 mmol/L, two concentrations that have no toxicological effects on NHA (Figure S1). These results indicated that matrine potently arrested GBM cells and reduced cell viability.

### 3.2 Matrine induces cellular senescence in GBM cells

**Figure 2:**
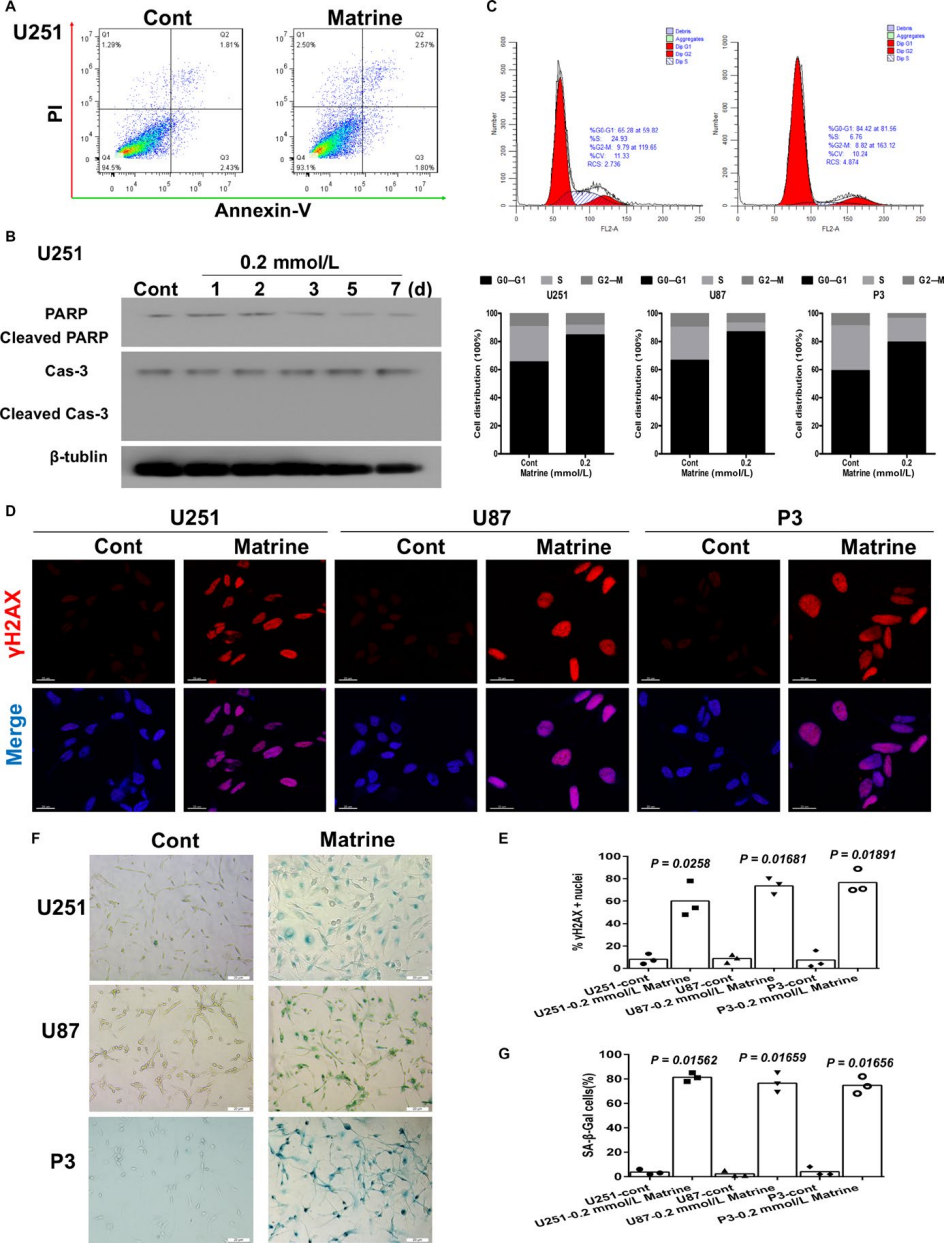
Matrine induces cellular senescence in GBM cells. A, Flow cytometric analysis of apoptosis in cells treated with 0.2 mmol/L matrine for 72 h as determined by annexin V‐ and/or FITC and propidium iodide staining for DNA content. The percentages of annexin V‐ and/or FITC‐positive cells are indicated. B, Western blot analysis of cas‐3, cleaved cas‐3, PARP, cleaved PARP, and β‐tublin expression in protein lysates (20 μg) prepared from U251 cells treated with matrine (0.2 mmol/L) for the indicated days. C, U251 cells in different phases of the cell cycle based on flow cytometric analysis (propidium iodide staining) of cells treated with 0.2 mmol/L matrine for 72 h. All data are expressed as the mean ± the SD of values from experiments performed in triplicate. D, Immunofluorescence staining for γH2AX (red) used to detect DNA damage and abnormal chromatin accumulated in U251 cells, U87, and P3 cells treated with matrine (0.2 mmol/L) for 72 h. Cell nuclei were counterstained with DAPI (blue). Scale bars = 20 μm. E, Graphic representation of cell number and γH2AX‐positive content U251, U87, and P3 cells treated with 0.2 mmol/L matrine for 72 h. Statistical analyses were performed using permutation test. *P*‐value came from the comparison between treated group and control group. F, In situ SA‐β‐gal assay to detect senescent cells. U251, U87, and P3 cells were treated with 0.2 mmol/L marine for 72 h. Cellular senescence was examined by SA‐β‐gal staining. Scale bars = 20 μm. G, Graphic representation of the percentage of SA‐β‐gal‐positive cells determined in four random fields per sample. All data are expressed as the mean ± the SD of values from experiments performed in triplicate. Statistical analyses were performed using permutation test. *P*‐value came from the comparison between treated group and control group

Chemotherapeutic agents lead to decreases in cancer cell proliferation through a variety of biological processes, including apoptosis and cellular senescence. In the case of matrine, studies have shown that the molecule induces apoptosis in a variety of cancer cell types.^16^, ^17^, ^18^ We therefore investigated first whether matrine induced apoptosis in GBM cells. Based on propidium iodide and annexin V staining and the TUNEL assay, the percentage of apoptotic cells did not increase significantly for either cell line treated with matrine (Figures 2A and S2A,C). Moreover, the levels of two key proteins involved in apoptosis, caspase 3, and PARP, remained unchanged as assessed on Western blot (Figures 2B and S2B). These results together indicated that the inhibitory effect of matrine on U251, U87, and P3 cells was not mediated through the induction of apoptosis.

Cellular senescence is a stable state of proliferative arrest that provides a barrier against malignant transformation.^19^ We therefore investigated senescence as an alternative cell fate in U251 and U87 cells treated with matrine. Flow cytometric analysis revealed that a greater percentage of U251, U87, and P3 cells were arrested in G1/G0 (~ 60% vs ~ 80% and 100%, control vs U251, U87, and P3 under 0.2 mmol/L matrine for 3 days; Figures 2C and S3A). Matrine treatment for 3 days also led to increased γH2AX‐positive nuclei indicative of DNA double‐strand breaks (Figure 2D,E), and accumulation of SA‐β‐gal‐positive cells (Figure 2F,G). Matrine treatment at 0.2 mmol/L did not induce cellular senescence in NHA (Figure S3B,C). Thus, cellular senescence is a mechanism underlying matrine‐induced inhibition of cell growth in vitro that might be independent of the tumor‐suppressing functions of PTEN.

### 3.3 Matrine induces cellular senescence by upregulating p27

**Figure 3:**
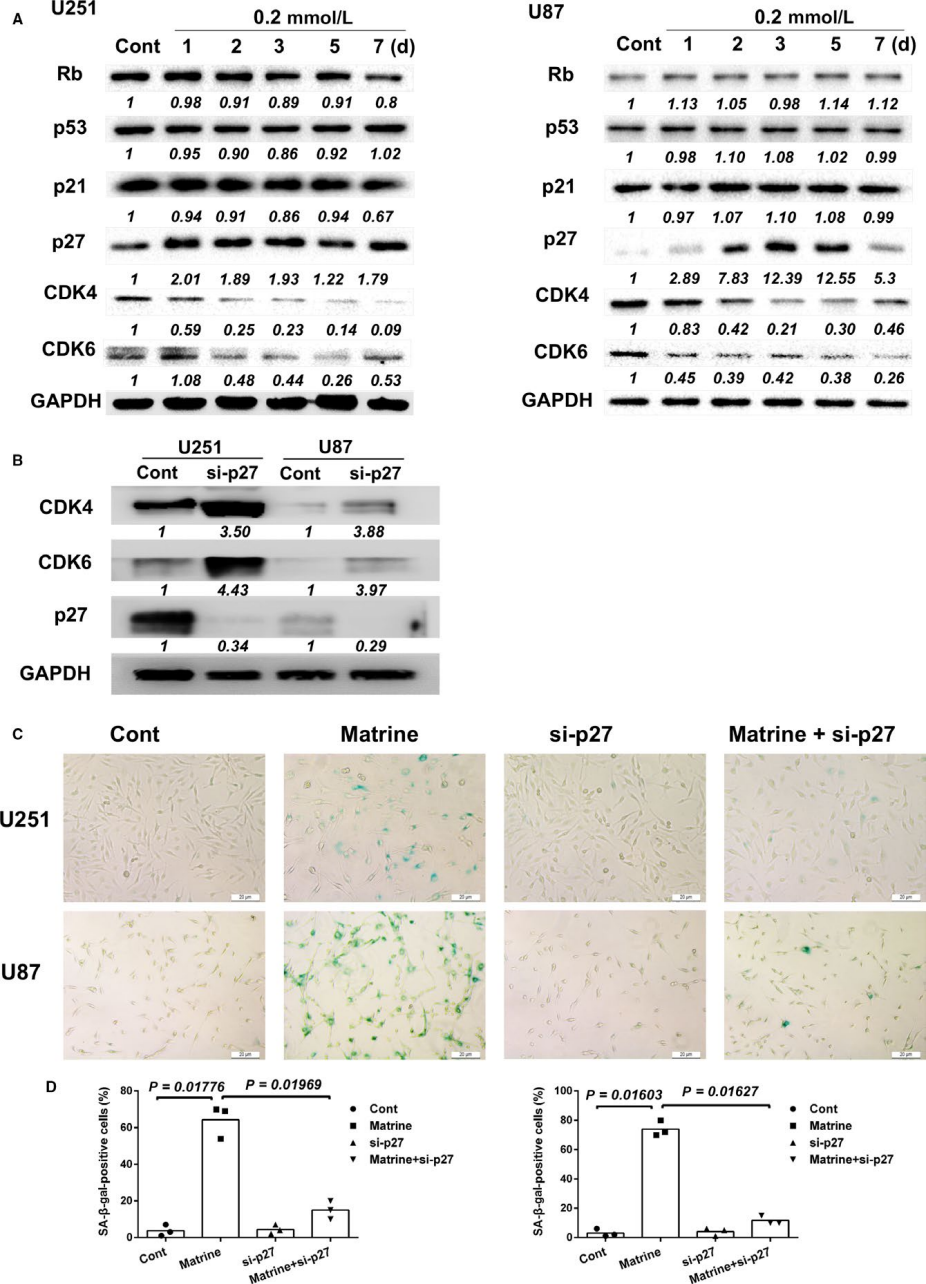
Matrine induces cellular senescence by upregulating p27. A, Western blot analysis for cell cycle‐associated proteins in lysates prepared from U251 and U87 cells treated with matrine for the indicated number of days. The immunoblots are representative of at least two independent experiments with GAPDH serving as a protein loading control. B, Western blot analysis of p27, CDK4, and CDK6 expression in U251 and U87 cells transfected with p27 siRNA for 48 h. C, In situ SA‐β‐gal assay to detect senescent cells. Cells were treated with 0.2 mmol/L matrine or p27 siRNA for 72 h and compared to controls. Scale bars = 20 μm. D, Graphic representation of the percentage of SA‐β‐gal‐positive cells determined in four random fields per sample. All data are expressed as the mean ± the SD of values from experiments performed in triplicate. Statistical analyses were performed using permutation test. *P*‐value came from the comparison between treated group and control group or between two treated groups

Senescence is a process triggered by cellular stress, leading to a permanent withdrawal from cell cycle through regulatory proteins such as Rb, p53, p21, and p27. To examine a potential role for these proteins in establishing cell cycle arrest, we first used Western blot analysis to determine expression levels in matrine‐treated cells. We treated cells with 0.2 mmol/L matrine and prepared lysates at approximately one‐day intervals for up to 7 days. Matrine treatment induced a marked increase in p27 protein expression in both U251 and U87 cells within 24 hours (>2×). In U87 cells, p27 levels continued to increase reaching a maximum at 3 days (7.83×; Figure 3A). Rb, p53, and p21 protein levels, however, remained unchanged over the course of the 7‐day treatment (Figure 3A). In contrast, the expression of cell cycle kinases downstream of p27, CDK4, and CDK6 was decreased after matrine treatment (Figure 3A).

To confirm the role of p27 in matrine‐induced senescence, we performed siRNA knockdown experiments in matrine‐treated GBM cells in vitro. Knockdown of p27 rescued GBM cells from matrine‐induced senescence (Figure 3B‐D). The percentage of SA‐β‐gal‐positive cells was reduced in si‐p27 transfected U251 and U87 cells by ~ 50% in the presence of 0.2 mmol/L matrine for 3 days. Taken together, these results indicated that matrine induces cellular senescence through upregulation of p27.

### 3.4 Matrine inhibits the PI3K/AKT/p27 signaling pathway

**Figure 4:**
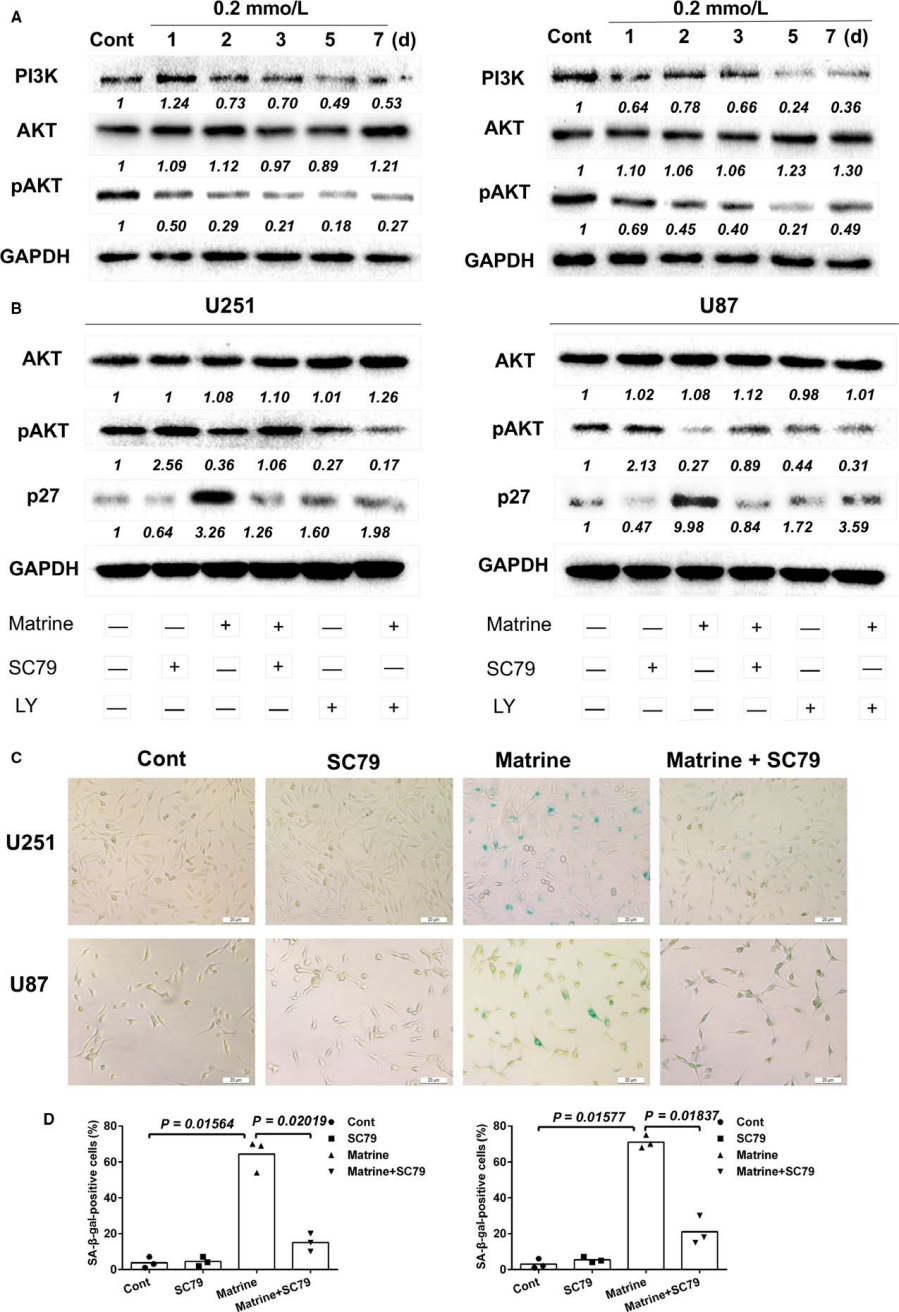
Matrine inhibits the PI3K/AKT/p27 signaling pathway. A, Western blot analysis for PI3K, AKT, pAKT, and GAPDH protein levels in protein lysates (20 μg) prepared from U251 and U87 cells treated with matrine for the number of days indicated. B, Western blot analysis to detect levels of AKT, pAKT, P27, and GAPDH in lysates prepared from U251 and U87 cells treated with matrine (0.2 mmol/L), AKT activator SC79 (5 μg/mL), or PI3K inhibitor LY294002 (1 mmol/L) 72 h. C, In situ SA‐β‐gal assay to detect senescent cells. Cells were treated with 0.2 mmol/L matrine and AKT activator SC79 for 72 h. Scale bars = 20 μm. D, Graphic representation of the percentage of SA‐β‐gal‐positive cells determined in four random fields per sample. All data are expressed as the mean ± SD of values from experiments performed in triplicate. Statistical analyses were performed using permutation test. *P*‐value came from the comparison between treated group and control group or between two treated groups

To further investigate the molecular mechanisms mediating matrine‐induced growth inhibition, we first used the Bioinformatics Analysis Tool for Molecular Mechanism (BATMAN) to identify potential signaling pathways. BATMAN is a bioinformatics tool developed to aid in the prediction of the molecular pathways and targets involved in the activities of traditional Chinese medicine. From the results of our analysis, we discovered a potential relationship between matrine treatment and the proteins PTEN and AKT of the PI3K‐AKT pathway (Figure S4). As this pathway is critical in the regulation of cellular senescence,^20^, ^21^ we tested whether PI3K/AKT signaling was involved in the response to matrine in GBM cells. Matrine treatment led to decreased expression of two proteins associated with cycling cells, PI3K and pAKT (~3× in 7 days; Figure 4A). These results indicated that reduced PI3K/AKT signaling might lead to the establishment of senescence in the presence of the alkaloid. We further investigated the role of PI3K and pAKT in the induction of matrine‐induced cellular senescence through exposure of cells to small molecules activating or inhibiting the pathway. GBM cells were treated with matrine and SC79, an activator of pAKT. Activation of the pathway was partially restored when cells were treated simultaneously with SC79; pAKT was partially increased (>2×) while p27 was decreased to control levels. The number of senescent SA‐β‐gal cells induced by matrine was correspondingly reduced in the presence of SC79 (~ 70% vs ~ 20%, matrine vs matrine + SC79; Figure 4B,C). Finally, because U87 and U251 are mutated in *PTEN*, we also examined the effects of matrine on LN18 and LN229, which are GBM cell lines wild type in *PTEN*. We found that in both LN18 and LN229, cells became senescent in the presence of matrine (Figure S5). These results all together indicated that the PI3K/AKT/p27 signaling pathway mediated matrine‐induced cellular senescence in U251 and U87 cells.

### 3.5 Downregulation of IGF1 enhances matrine‐induced senescence in GBM cells

**Figure 5:**
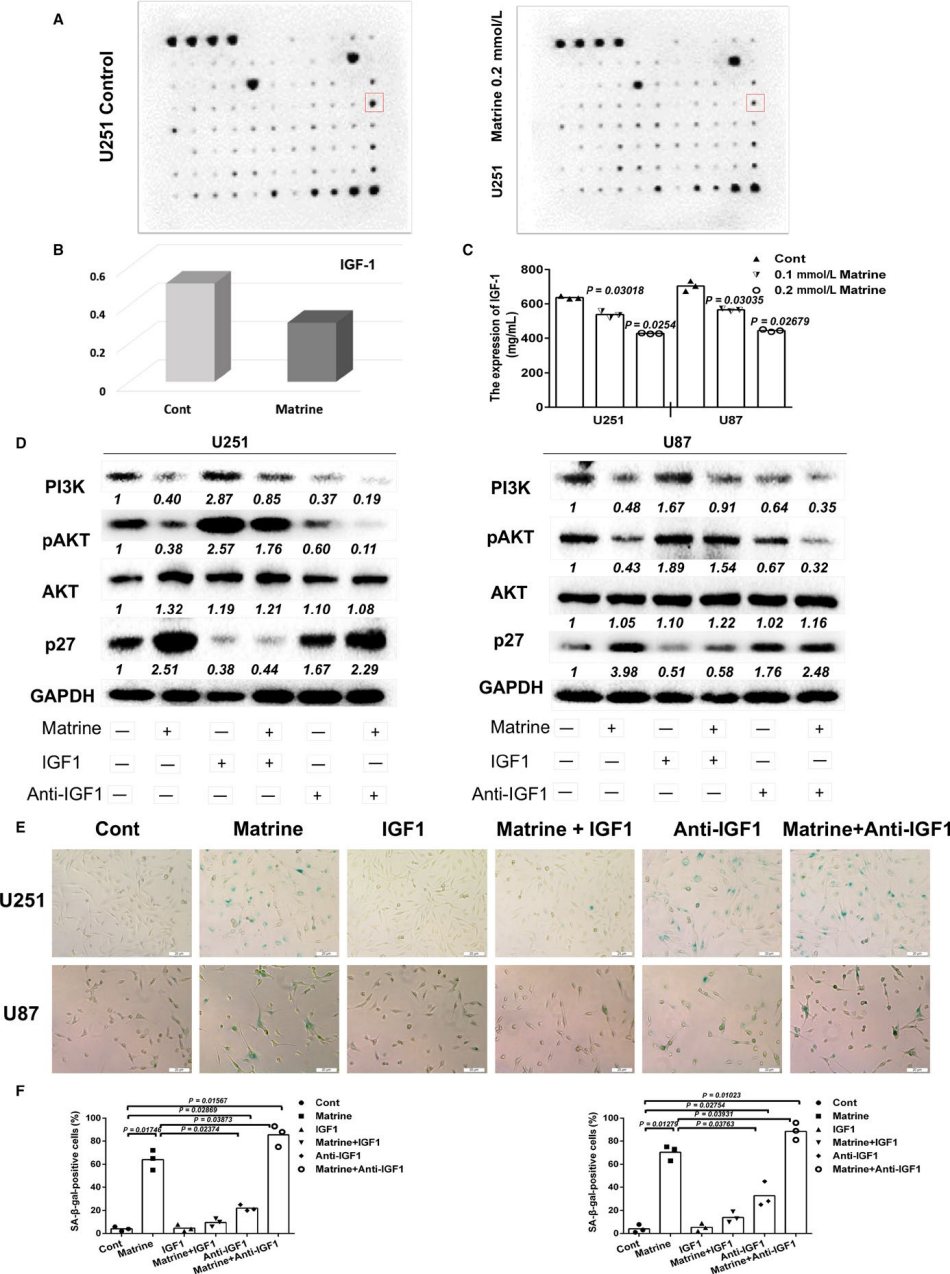
Downregulation of IGF1 enhances matrine‐induced cellular senescence in GBM cells. A, Cytokine array to detect secreted protein levels in controls and matrine‐treated U251 for 72 h. B, Fold expression levels of IGF1 as determined in the cytokine assay. Chemiluminescent signals quantified with ImageJ software. C, Results from ELISA performed to detect levels of IGF1 in media collected from control and matrine‐treated U251 and U87 cells. Statistical analyses were performed using permutation test. *P*‐value came from the comparison between treated group and control group. D, Western blot analysis of PI3K, pAKT, AKT, p27, and GAPDH expressions in U251 and U87 cells treated with 0.2 mmol/L matrine, exogenous IGF1 (200 ng/mL), or IGF1 antibody (1 mg/mL) for 72 h. E, In situ SA‐β‐gal assay to detect senescent cells. Cells were treated with matrine, exogenous IGF1, or IGF1 antibody for 72 h. Scale bars = 20 μm. F, Graphic representation of the percentage of SA‐β‐gal‐positive cells as determined in four random fields per sample. All data are expressed as the mean ± the SD of values from experiments performed in triplicate. Statistical analyses were performed using analysis of variance in randomized blocks. *P*‐value came from the comparison between treated group and control group or between two treated groups

Cellular senescence is a phenomenon of prolonged cell cycle arrest. Senescent cells are still metabolically active but display significant differences in protein expression from their cycling counterparts which are referred to as a senescence‐associated secretory phenotype (SASP). A variety of soluble proteins, including cytokines, chemokines, and growth factors, are affected, and ultimately influence processes, such as tumor suppression, tumor promotion, aging, and tissue repair. We therefore used array analysis to screen for secreted cytokines that might be altered in GBM cells after matrine treatment. Senescence was induced in U251 cells through treatment with 0.2 mmol/L matrine, and cell supernatants were collected after 3 days and incubated with the arrays. The analysis revealed an ~3× reduction in secreted IGF1 from matrine‐induced senescent U251 cells compared to controls (Figure 5A‐C).

IGF1 promotes PI3K/AKT signaling, and therefore cell survival and proliferation.^22^ To examine how increased IGF1 might influence the establishment of senescence, anti‐IGF1 antibody or recombinant IGF1 was added with matrine to GBM cells, and cells were collected to prepare protein lysates for Western blot analysis 3 days later. Blocking of IGF1 led to increased expression of p27 in U251 and U87 cells (~ 2 × ; Figure 5D). PI3K and pAKT, however, were decreased (Figure 5D). Furthermore, ~ 28% of U251 and ~ 32% of U87 cells became senescent after 5 days of treatment with anti‐IGF1 (1 mg/mL; Figure 5E,F). In contrast, exogenous IGF1 partially reversed the expression of PI3K, pAKT, and p27 (Figure 5D). Finally, cotreatment of cells with matrine and anti‐IGF1 or exogenous IGF1 led to changes in the percentage of senescent cells. Anti‐IGF1 with matrine was more effective at inducing cellular senescence in both U251 and U87 cells than treatment with matrine alone (~ 20% increase, *P *<* *0.05; Figure 5E and 5F). The number of SA‐β‐gal‐positive cells induced by matrine, however, decreased when IGF1 was added to the medium. Taken together, these results indicated that blocking IGF1 enhanced matrine‐induced senescence in glioma cells.

### 3.6 Matrine inhibits growth and induces cellular senescence of GBM cells in vivo

**Figure 6:**
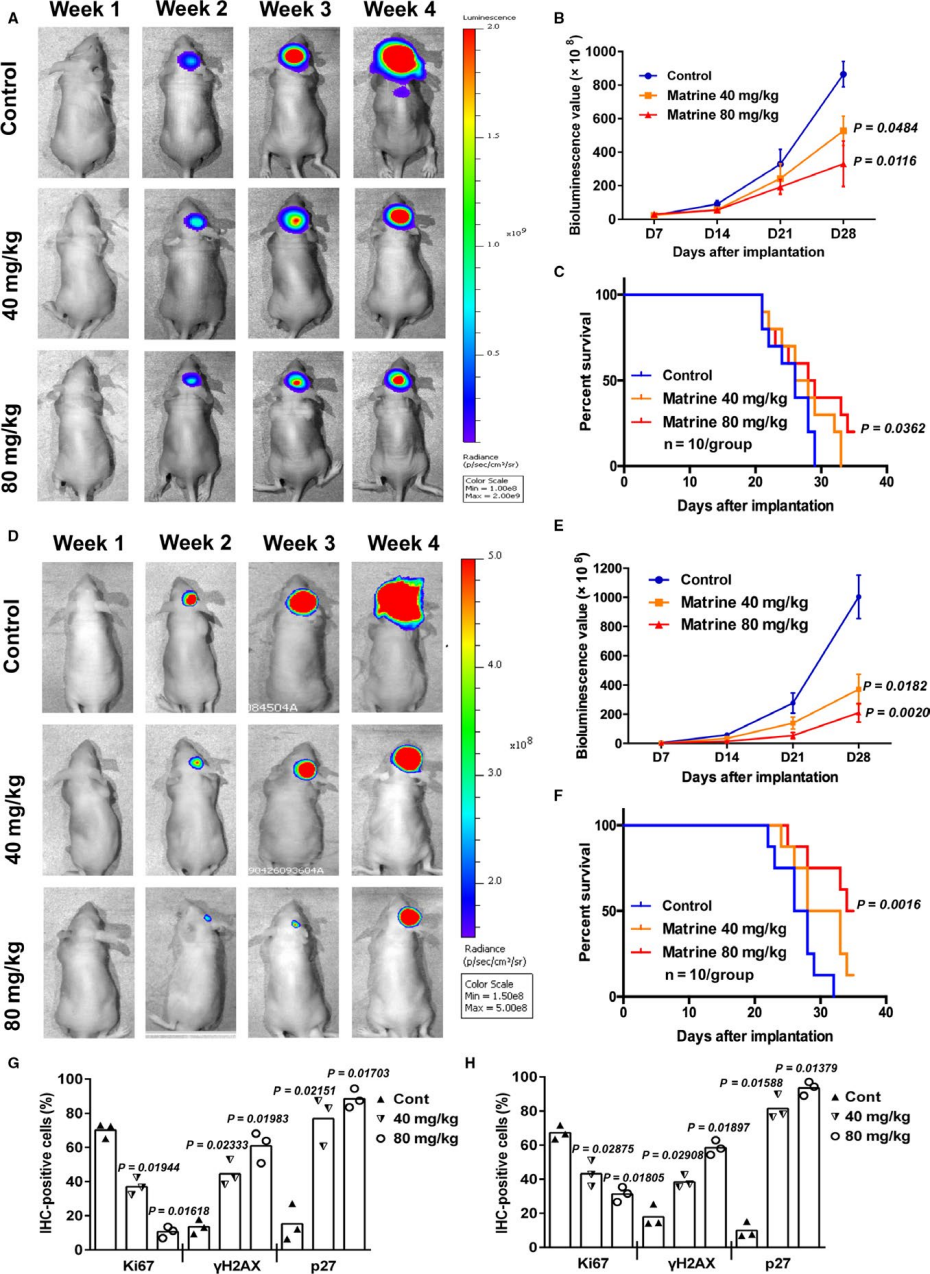
Matrine inhibits growth and induces cellular senescence of U251 GBM cells in vivo. A, U251 cells expressing luciferase were orthotopically implanted into athymic nude mice. Tumor growth was monitored using the IVIS‐200 imaging system for detection of bioluminescence. Bioluminescent signals were measured at days 7, 14, 21, and 28 after implantation. B, Bioluminescence values plotted as a function of time in days to assess tumor growth (days 7, 14, 21, and 28). Statistical analyses were performed using permutation test. *P*‐value came from the comparison between treated group and control group. C, Overall survival as determined by Kaplan‐Meier survival curves. A log‐rank test was used to assess the statistical significance of the differences (*P* < 0.05). D, P3 cells expressing luciferase were orthotopically implanted into athymic nude mice, and tumor growth was monitored using the IVIS‐200 imaging system for detection of bioluminescence. Bioluminescent signals were measured at days 7, 14, 21, and 28 after implantation. E, Bioluminescence values plotted as a function of time in days to assess tumor growth (days 7, 14, 21, and 28). Statistical analyses were performed using permutation test. *P*‐value came from the comparison between treated group and control group. F, Overall survival as determined by Kaplan‐Meier survival curves. A log‐rank test was used to assess the statistical significance of the differences (*P* < 0.01). G, Graphic representation of percentage of Ki67, H2AX, and p27‐positive cells in control and matrine‐treated U251 xenografts as determined with immunohistochemistry. Statistical analyses were performed using permutation test. *P*‐value came from the comparison between treated group and control group. H, Graphic representation of percentage of Ki67, H2AX, and p27‐positive cells in control and matrine‐treated P3 xenografts as determined with immunohistochemistry. All data are expressed as the mean ± the SD of values from experiments performed in triplicate. Statistical analyses were performed using permutation test. *P*‐value came from the comparison between treated group and control group

The in vitro data shown above clearly demonstrated a potent inhibitory effect of matrine against GBM cells in vitro. To determine efficacy in vivo, orthotopic xenografts were established in mice and treated with matrine. Luciferase‐stable‐U251 or luciferase‐stable‐P3 were inoculated into the right striatum of mouse brains, and mice were i.p. injected with matrine (40 and 80 mg/kg body weight) every other day starting on day 3 following implantation for 4 weeks. Tumor growth was monitored over time using bioluminescence values. Matrine monotherapy significantly reduced tumor growth (~ 83 × 10^9^ vs ~ 51 × 10^9^ and ~ 26 × 10^9^ photons/s, control vs 40 mg/kg and 80 mg/kg matrine; Figure 6A,B,D,E). Kaplan‐Meier analysis of the survival data also demonstrated a statistically significant difference for overall survival between control and matrine‐treated animals (28 days vs >35 days, *P *<* *0.05; Figure 6C,F).

Immunohistochemistry was performed on sections from control and treated xenografts to confirm these results at the molecular level. Staining with Ki67, a marker for proliferation, indicated that the proliferation of glioma cells was greatly inhibited in matrine‐treated mice (Figures 6G,H and S6). In addition, the expression of p27 and γH2AX was significantly increased in xenografts from matrine‐treated mice relative to controls (p27: ~ 20% vs ~80%, control vs treated, *P *<* *0.05; Figures 6G,H and S6). These data corroborated the observations made in vitro and demonstrated that matrine therapy has promise as treatment for GBM in patients.

## 4 DISCUSSION

Our results demonstrated that the traditional Chinese medicine matrine possesses a potent antitumor effect against GBM cells in vitro and in vivo. We found that matrine elicits growth arrest in GBM cells, largely by inducing cellular senescence, and it does so importantly through the inhibition of PI3K/AKT signaling. Studies have demonstrated that cellular senescence is a natural barrier in the development of human cancer.^23^, ^24^ Induction of senescent‐like growth arrest has become a new strategy for anticancer treatment.^25^ Thus, these results have important implications for matrine in the treatment of GBM, particularly due to the fact that aberrant signaling of PI3K/AKT occurs in GBM through the frequent loss of the tumor suppressor gene *PTEN*.

We searched for a potential signaling pathway network for matrine on the BATMAN database, a bioinformatics tool developed specifically to facilitate the molecular understanding of the function of traditional Chinese medicines. We found a significant correlation between AKT, PTEN, and matrine treatment. PTEN plays a vital role as a cellular tumor suppressor and regulates PI3K/AKT signaling.^26^, ^27^ As PI3K/AKT is an important pathway in the regulation of cellular senescence,^28^, ^29^ we further tested whether PI3K/AKT contributed to senescence in response to matrine treatment. Matrine treatment led to decreased expression of PI3K and pAKT in GBM cells, and activation of the signaling pathway partially restored cell growth when matrine‐treated cells were simultaneously exposed to SC79, an activator of pAKT. Interestingly, application of LY294002, an inhibitor of PI3K, induced senescence in GBM cells, further supporting the idea that inhibition of the PI3K/AKT pathway underlies the induction of cellular senescence in U251 and U87 cells. These findings establish the PI3K/AKT signaling pathway is critical to the induction of senescence in matrine‐treated GBM cells.

Although cellular senescence is an irreversible arrest of cell proliferation, senescent cells remain metabolically active.^24^, ^30^ The expression profile of senescent cells, or the senescence‐associated secretory phenotype (SASP), is characterized and exhibits increased expression and secretion of various chemokines, cytokines, and other secretory proteins which are implicated in tumor progression and inflammatory responses.^23^ Matrine‐induced senescent U251 cells secreted lower levels of IL6, IGF1, angiogenin (ANG), TIMP2, and MIF and higher levels of CXXL10, FGF9, CK3CL1, MCP2, and TNFSF14. We subsequently demonstrated that inhibition of IGF1, which promotes proliferation in GBM,^31^ also induces cellular senescence in GBM cells in vitro. This result further supports the role of PI3K/AKT signaling in matrine‐induced senescence, as IGF1 is known to promote cell growth through this pathway. Finally, we were able to rescue cells from matrine‐induced cellular senescence through activated AKT. Matrine may inhibit secretion of IGF1 through a variety of mechanisms, such as through protein degradation or inhibition of transcription, for example, which we plan to investigate in the future. However, in this study, we conclude that matrine induces cellular senescence through the downregulation of the IGF1/PI3K/AKT pathway.

A potential weakness of the in vivo studies is the concentration of matrine used. Matrine is currently used as an anti‐hepatitis B drug, and it has been used in clinic for many years. However, the concentration we used in our experiments is higher than the dose of 150 mg used daily in the clinic for hepatitis B. The optimal dose for cancer treatment may require further analysis and refinement in future experiments.

**Figure 7:**
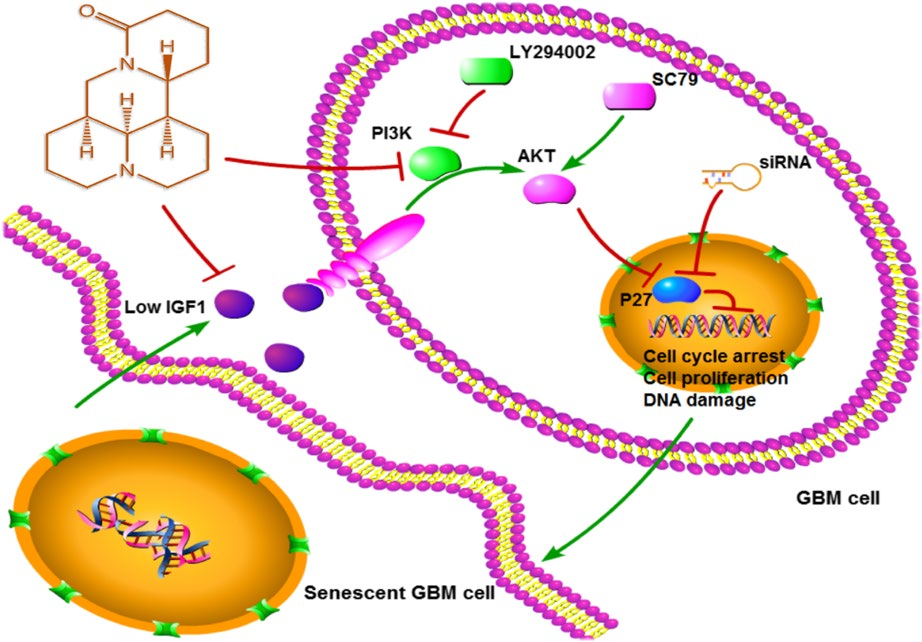
Proposed model for mechanism of matrine activity on human GBM cells. Matrine suppresses GBM cell growth and invasion by inhibiting PI3K/AKT/p27 signaling and inducing senescence. Decreased IGF1 in matrine‐induced senescent cells enhances response to matrine

p53, RB, p21, and p27 have well‐established and important roles in inducing senescence.^32^ On protein blots, we only observed significant changes in p27 in response to matrine. p27 is a regulator of cyclin‐dependent kinases (CDK); when bound, p27 inactivates CDKs and induces cell cycle arrest at G1, S, or G2 phases.^33^ Our data demonstrated that siRNA knockdown of p27 rescued GBM cells from matrine‐induced senescence and implicate p27 as a critical molecule mediating matrine‐induced cellular senescence. Thus, we were able to demonstrate that matrine inhibits GBM cell growth through several components along the same pathway, which is illustrated in Figure 7.

In summary, our data indicate that matrine inhibits growth of GBM cells by inducing cellular senescence. The alkaloid extract elicits growth arrest through the IGF1/PI3K/AKT/p27 signaling pathway which is frequently activated in GBM due to loss of *PTEN*. Matrine thus warrants further investigation as a natural bioactive molecule with cancer‐killing potential.

## CONFLICT OF INTEREST

The authors declare no conflict of interests.
