# Supplementary figures and images for: Matrine induces senescence of human glioblastoma cells through suppression of the IGF1/PI3K/AKT/p27 signaling pathway
Source: Cancer Med. 2018 Aug 5;7(9):4729–43. doi: 10.1002/cam4.1720 (PMC6143938; doi:10.1002/cam4.1720)

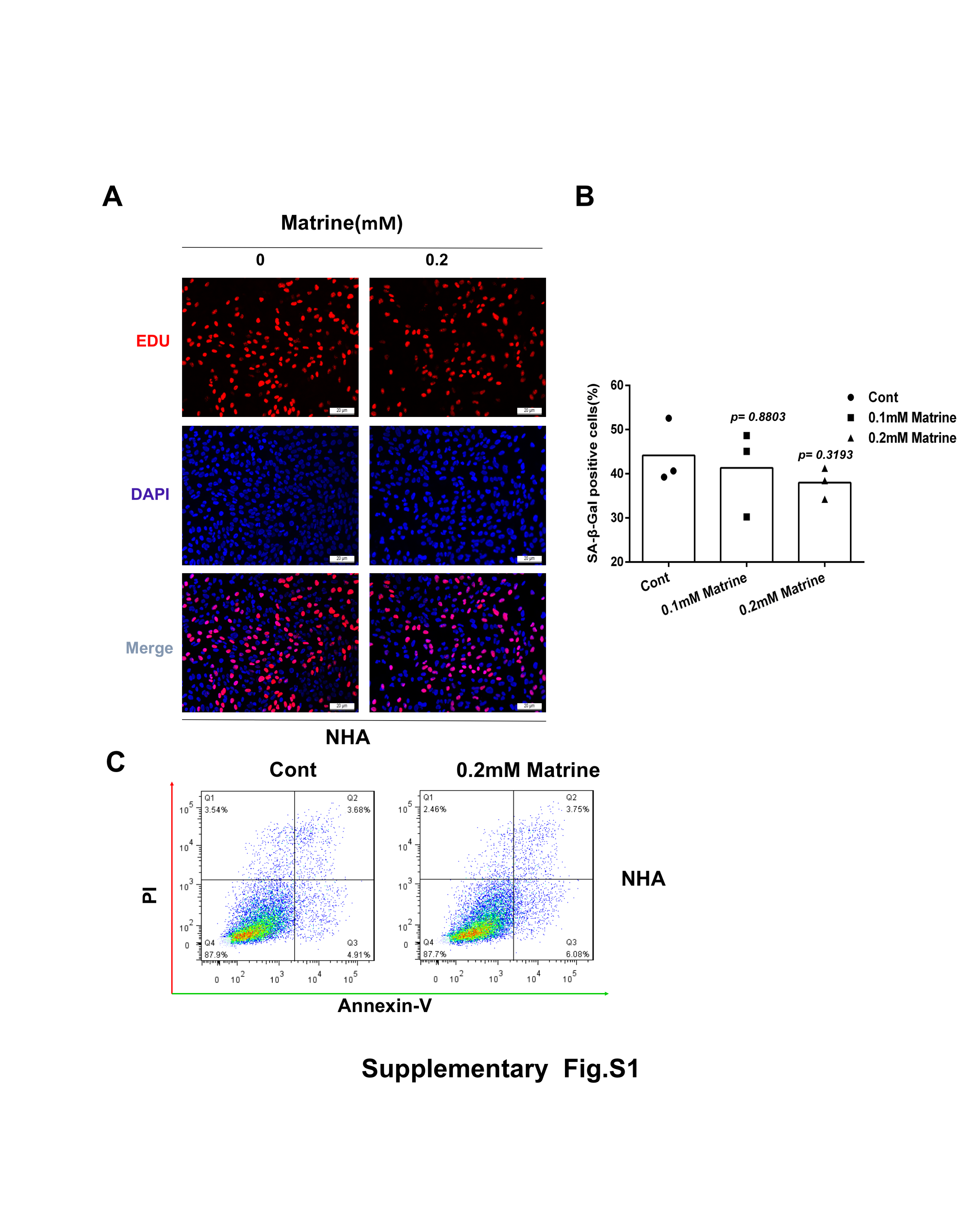

Supplement: Supplementary file 1 [file CAM4-7-4729-s001.tiff]

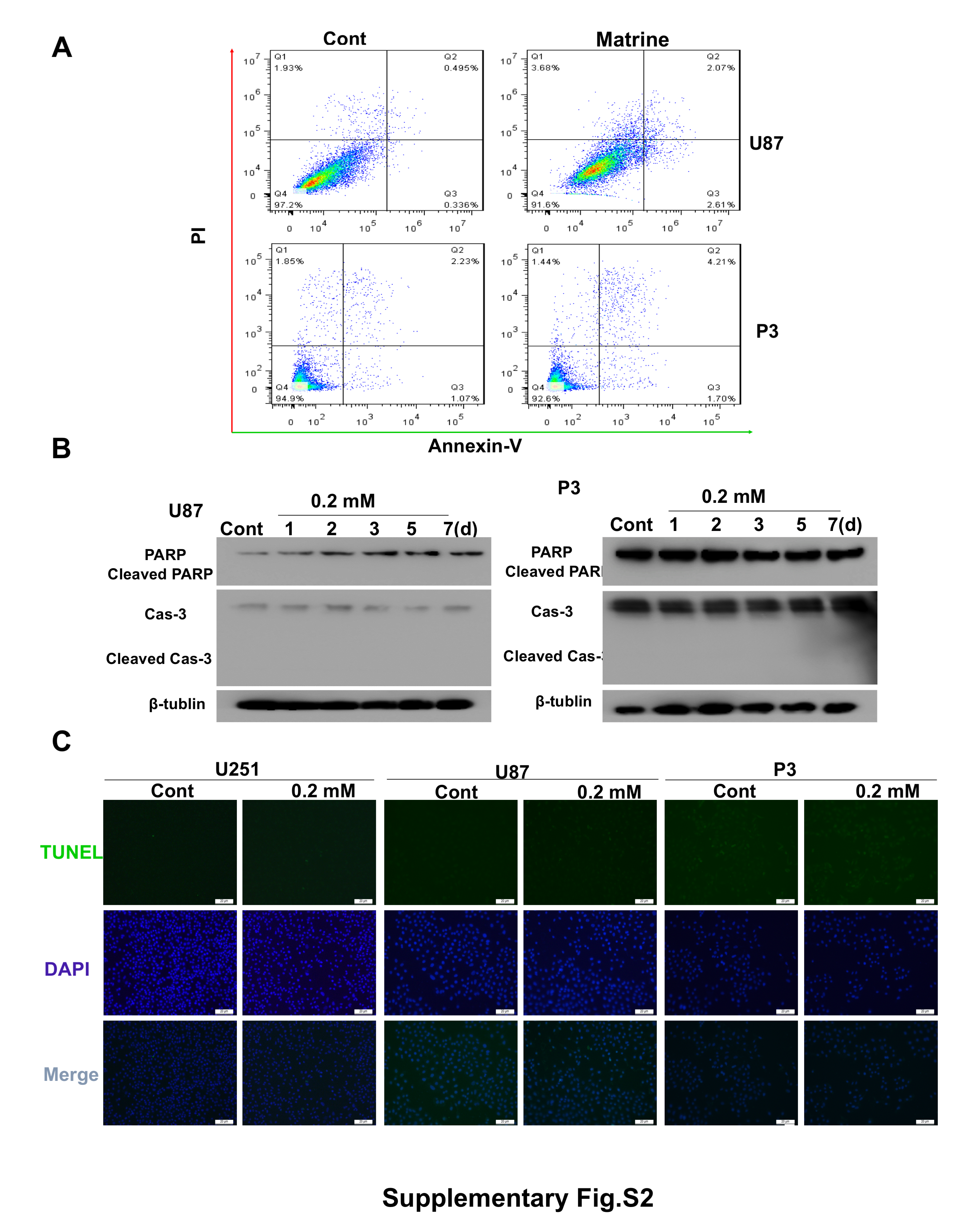

Supplement: Supplementary file 2 [file CAM4-7-4729-s002.tiff]

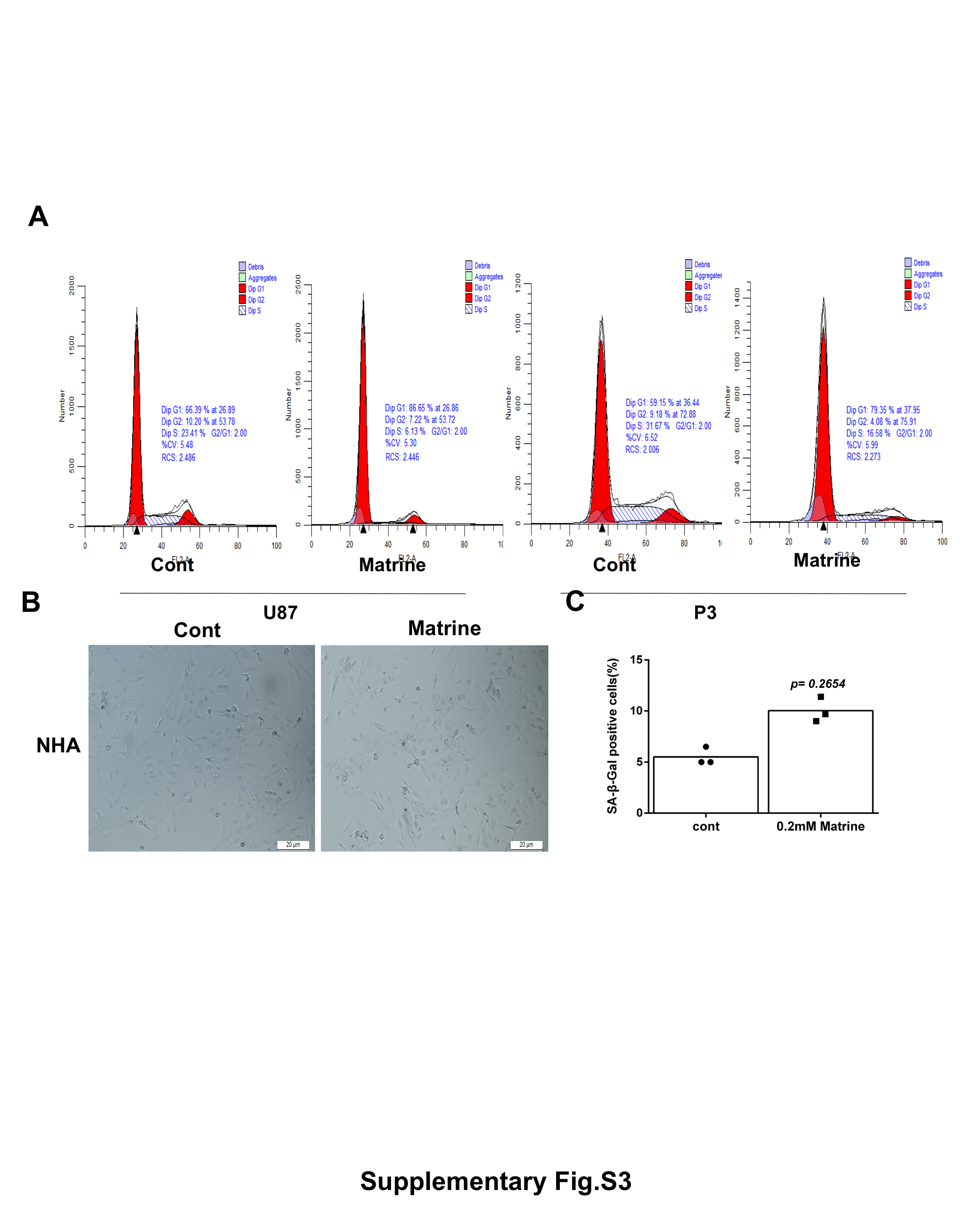

Supplement: Supplementary file 3 [file CAM4-7-4729-s003.tif]

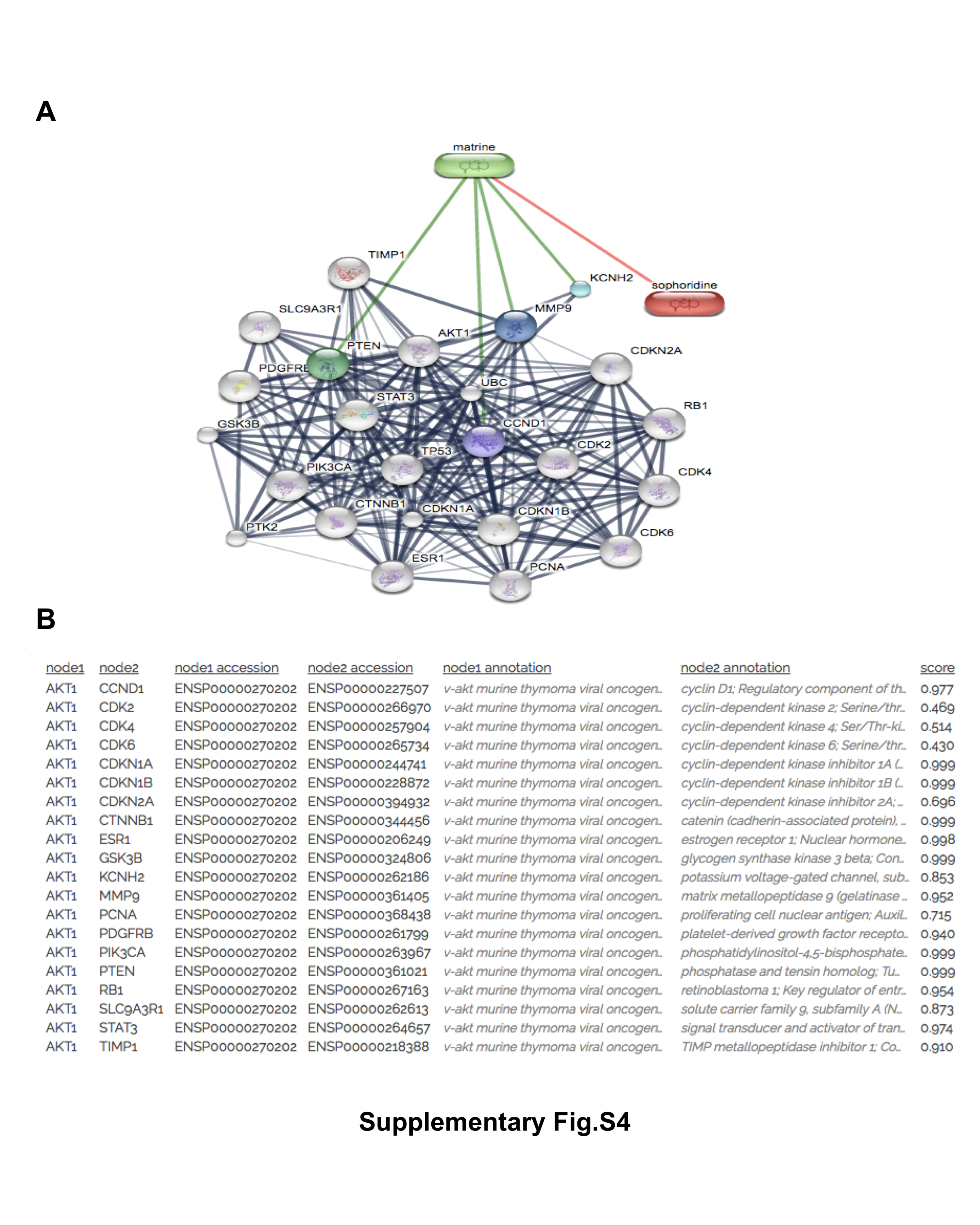

Supplement: Supplementary file 4 [file CAM4-7-4729-s004.tif]

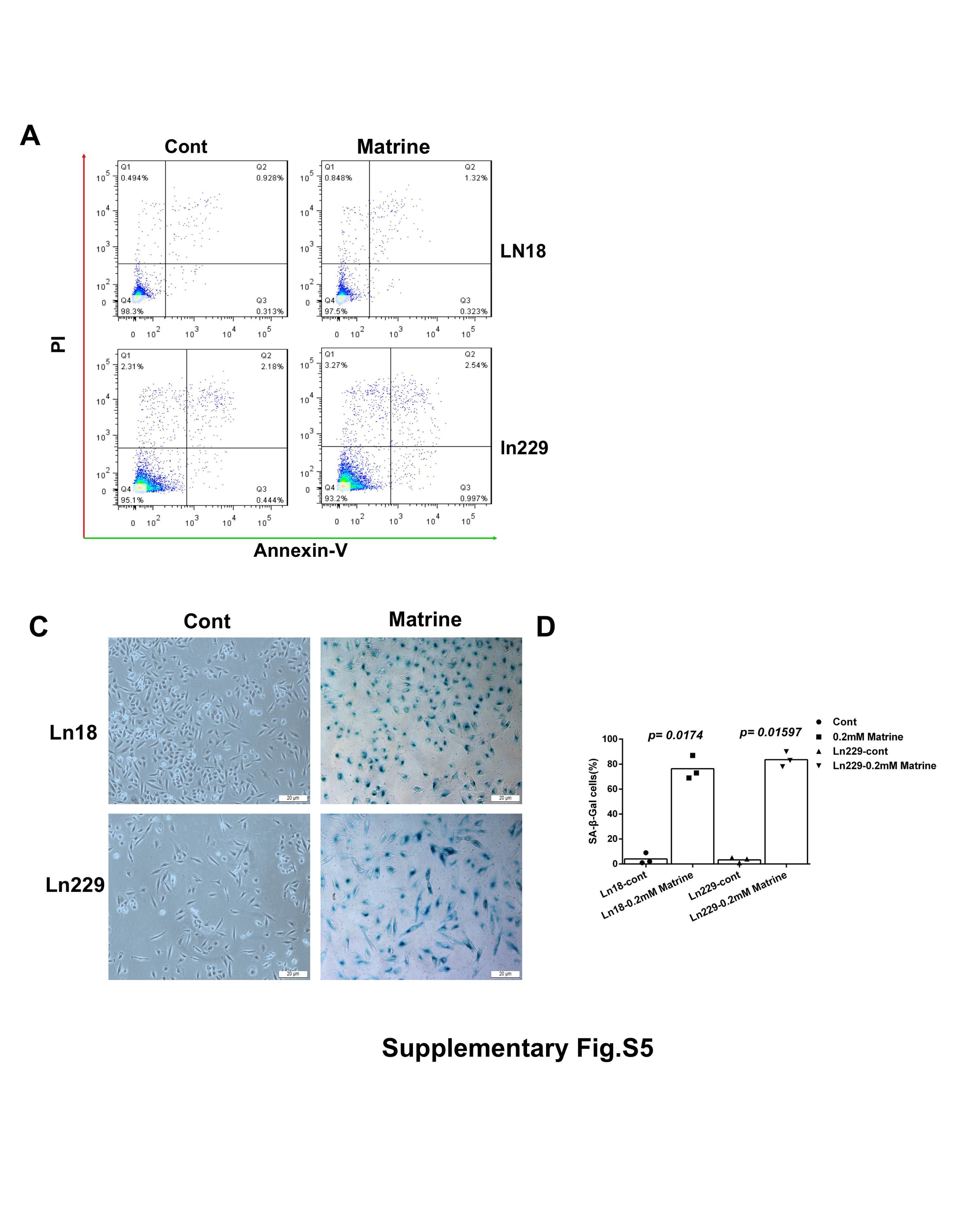

Supplement: Supplementary file 5 [file CAM4-7-4729-s005.tif]

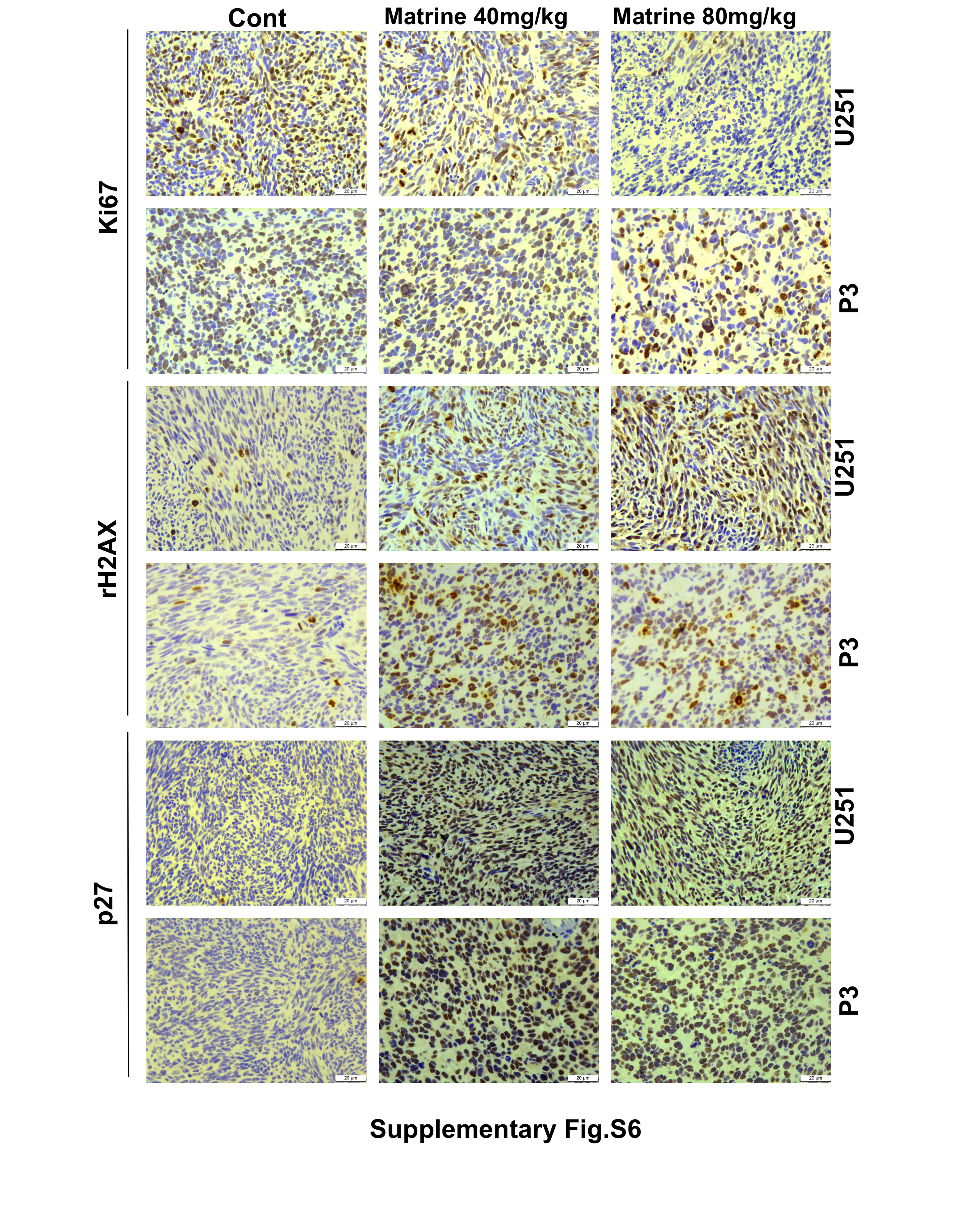

Supplement: Supplementary file 6 [file CAM4-7-4729-s006.tiff]

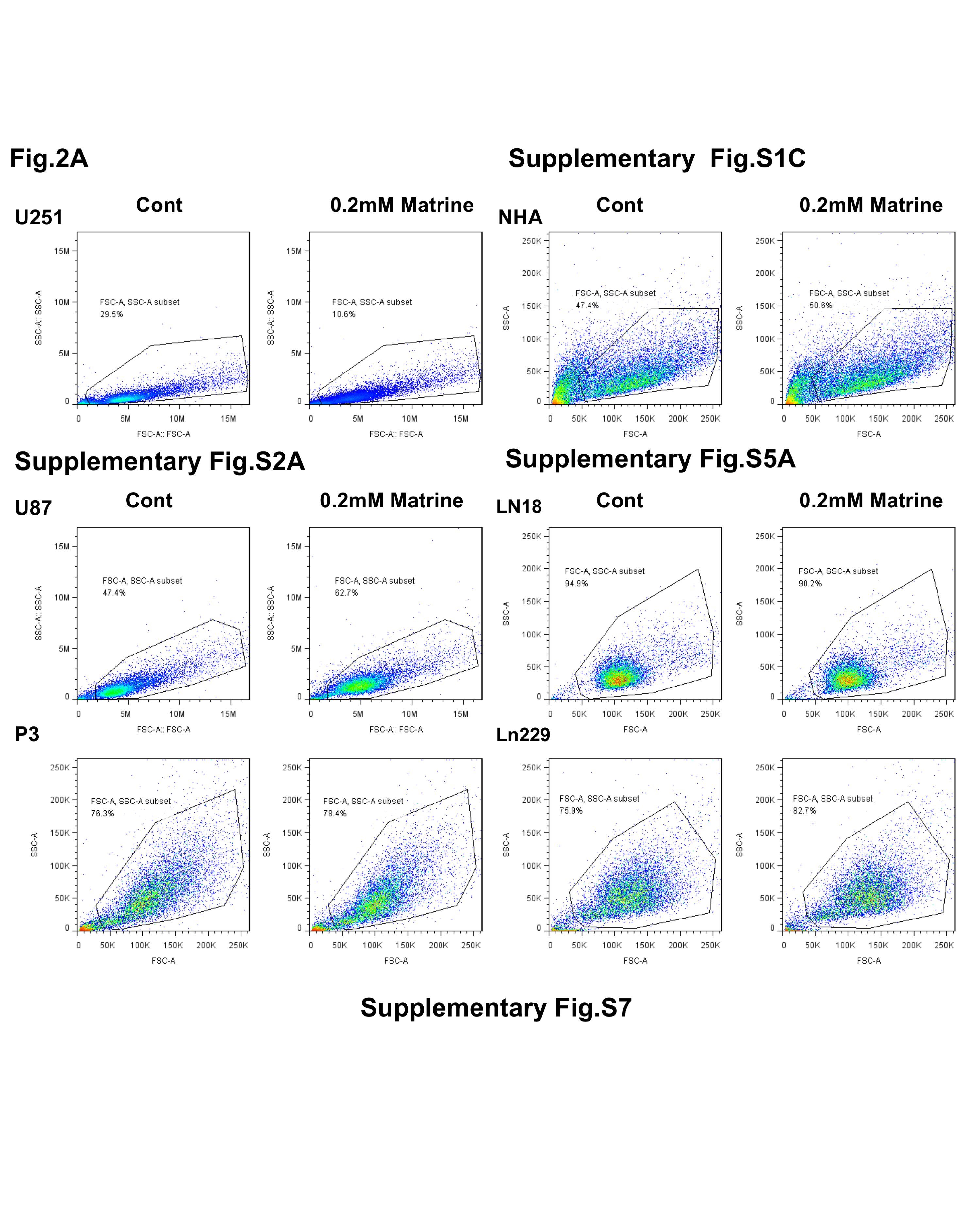

Supplement: Supplementary file 7 [file CAM4-7-4729-s007.tiff]

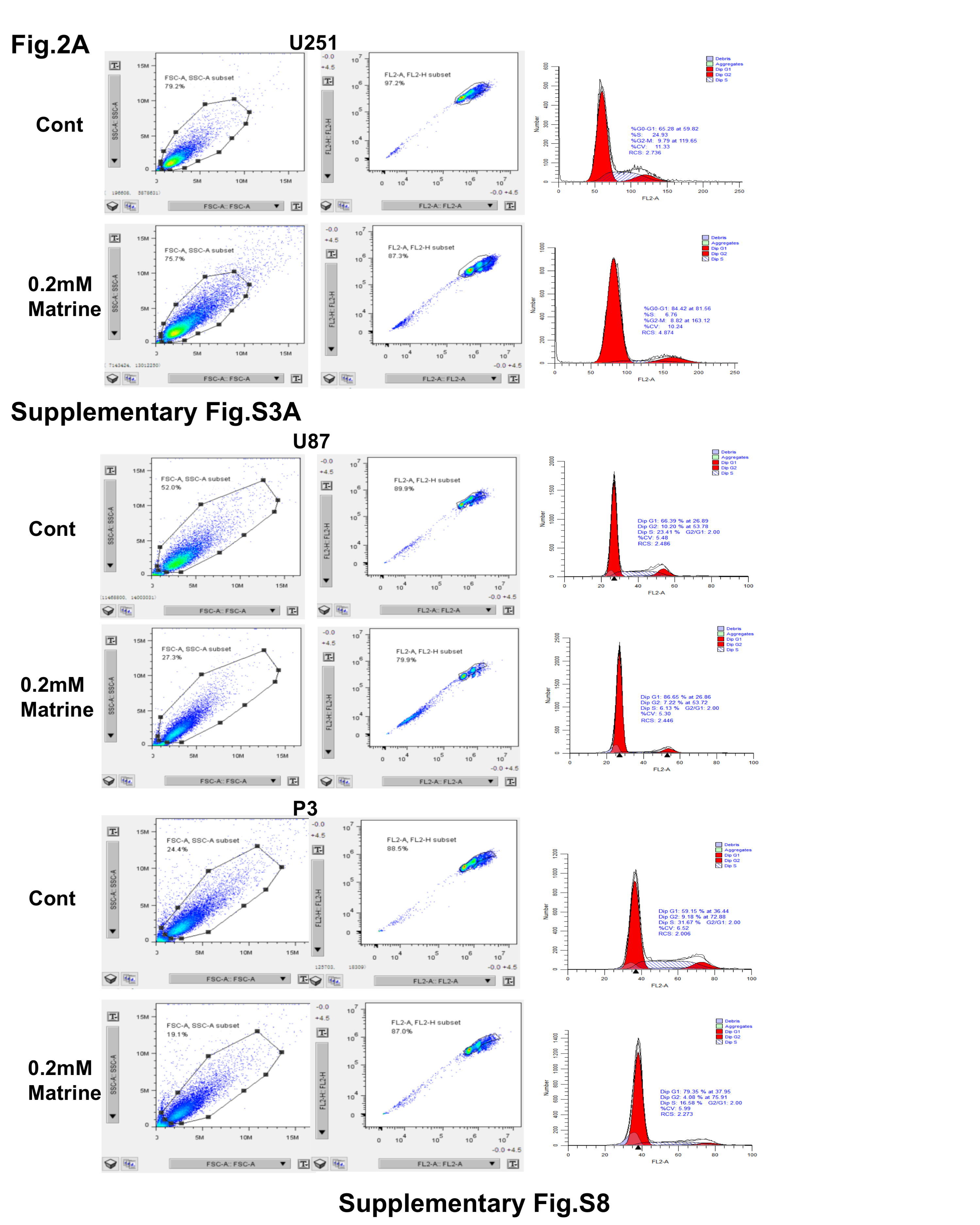

Supplement: Supplementary file 8 [file CAM4-7-4729-s008.tiff]

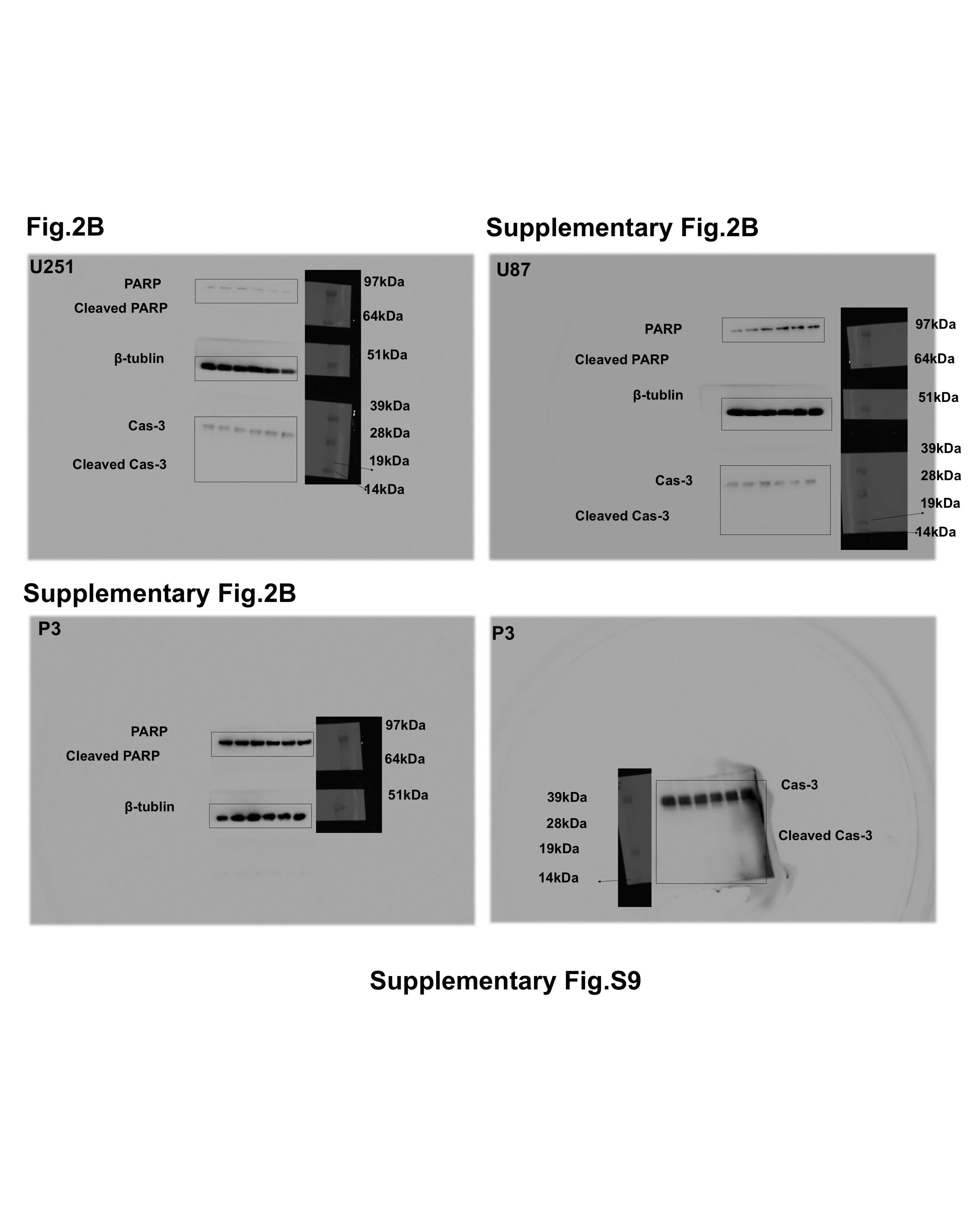

Supplement: Supplementary file 9 [file CAM4-7-4729-s009.tif]

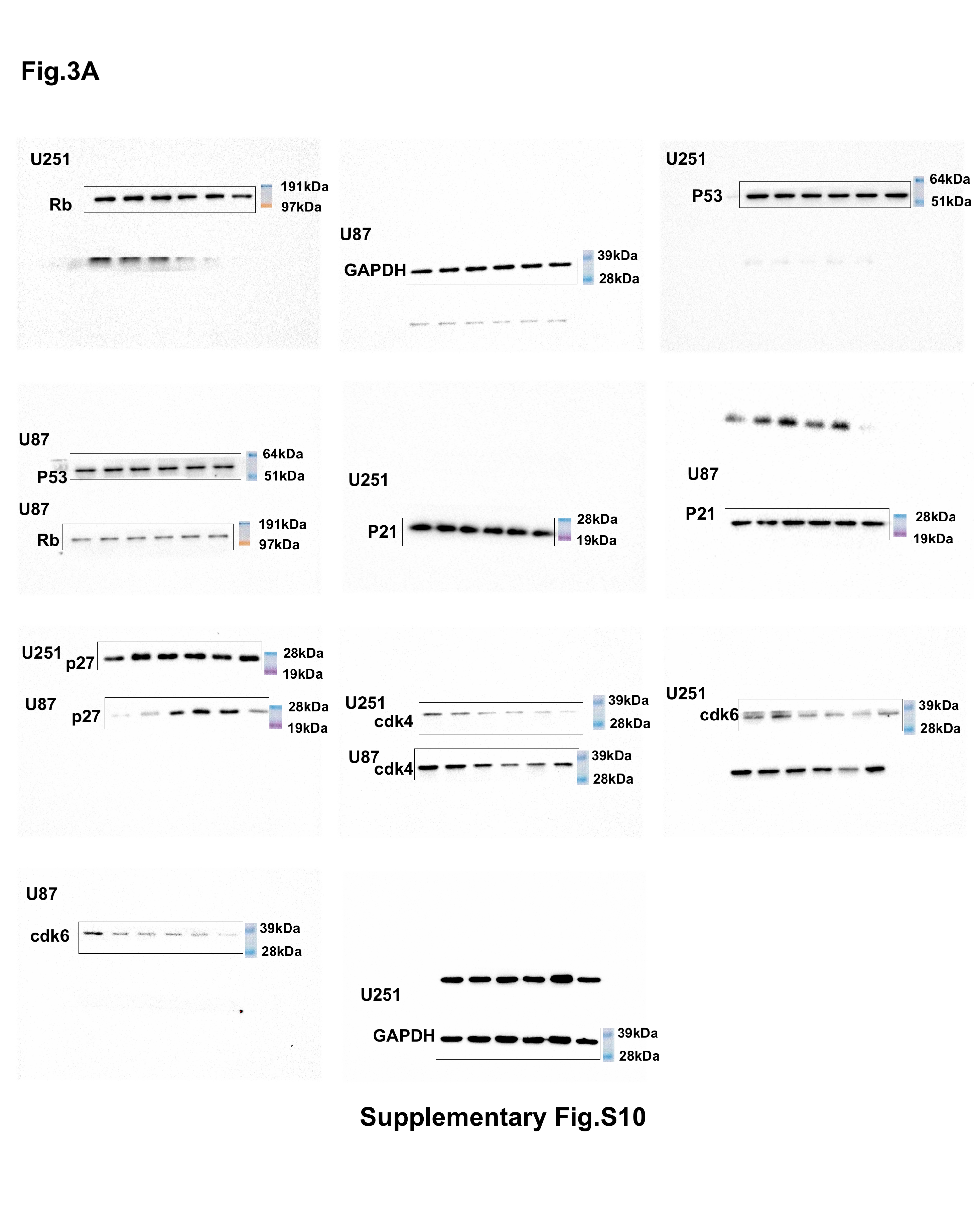

Supplement: Supplementary file 10 [file CAM4-7-4729-s010.tif]

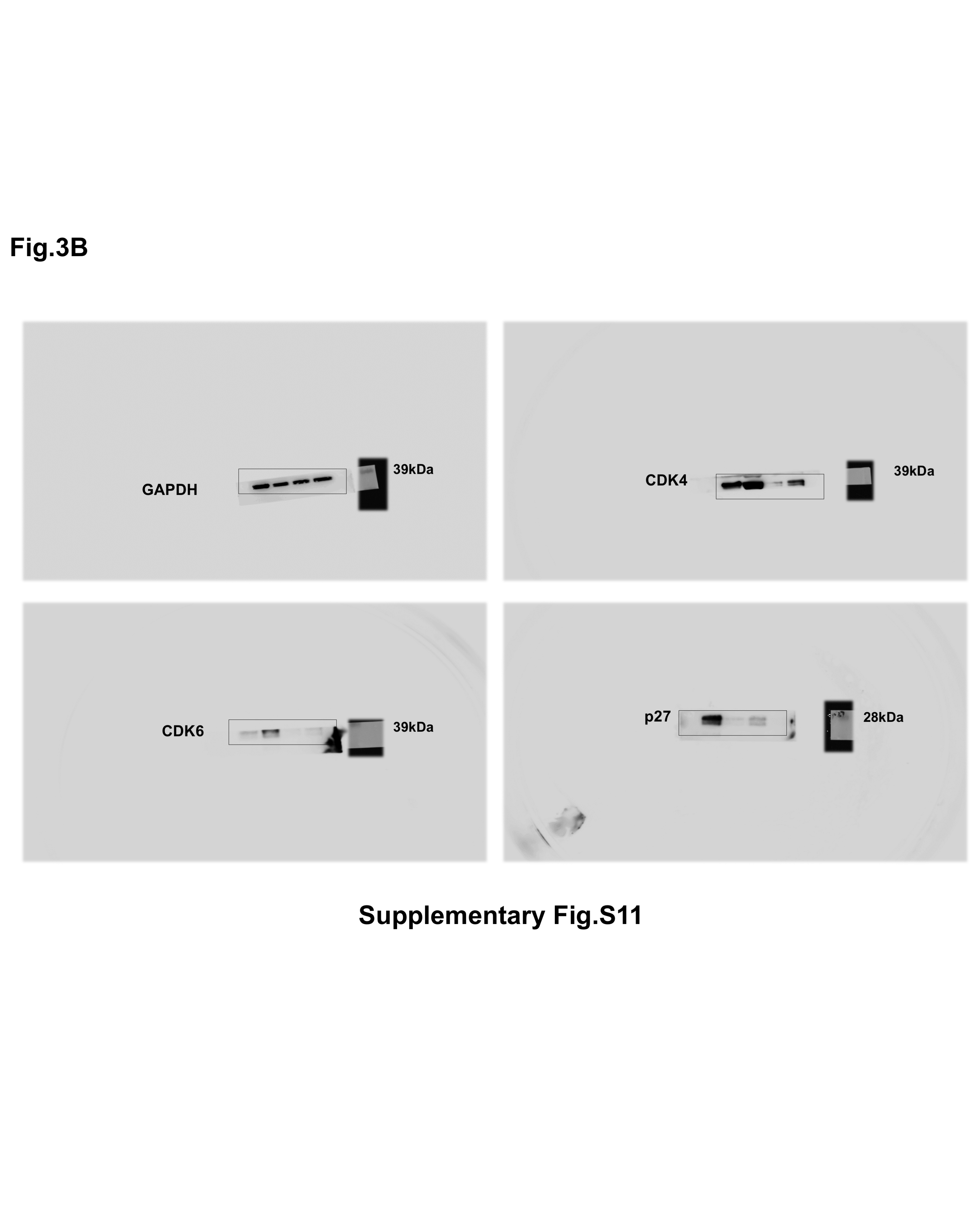

Supplement: Supplementary file 11 [file CAM4-7-4729-s011.tif]

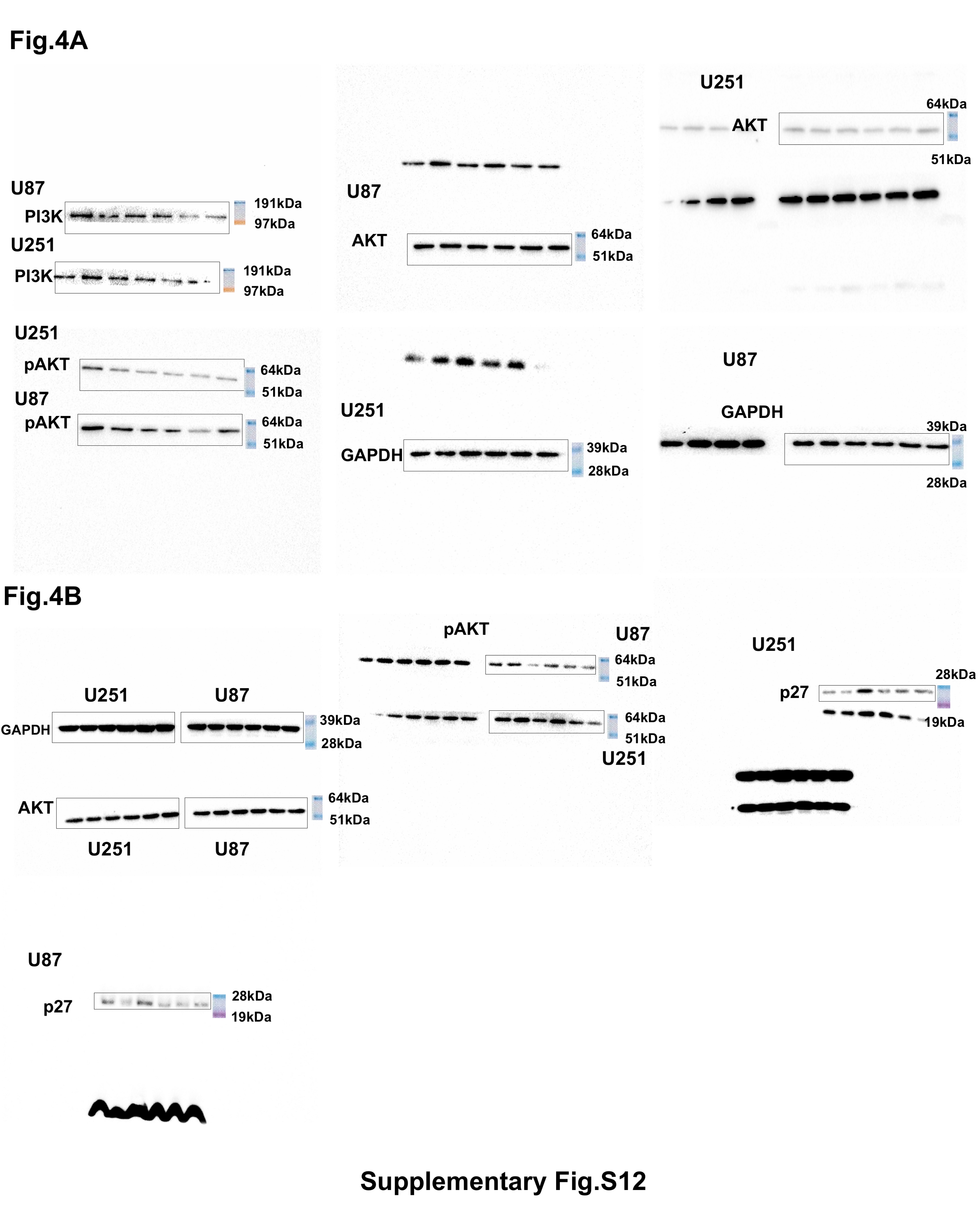

Supplement: Supplementary file 12 [file CAM4-7-4729-s012.tif]

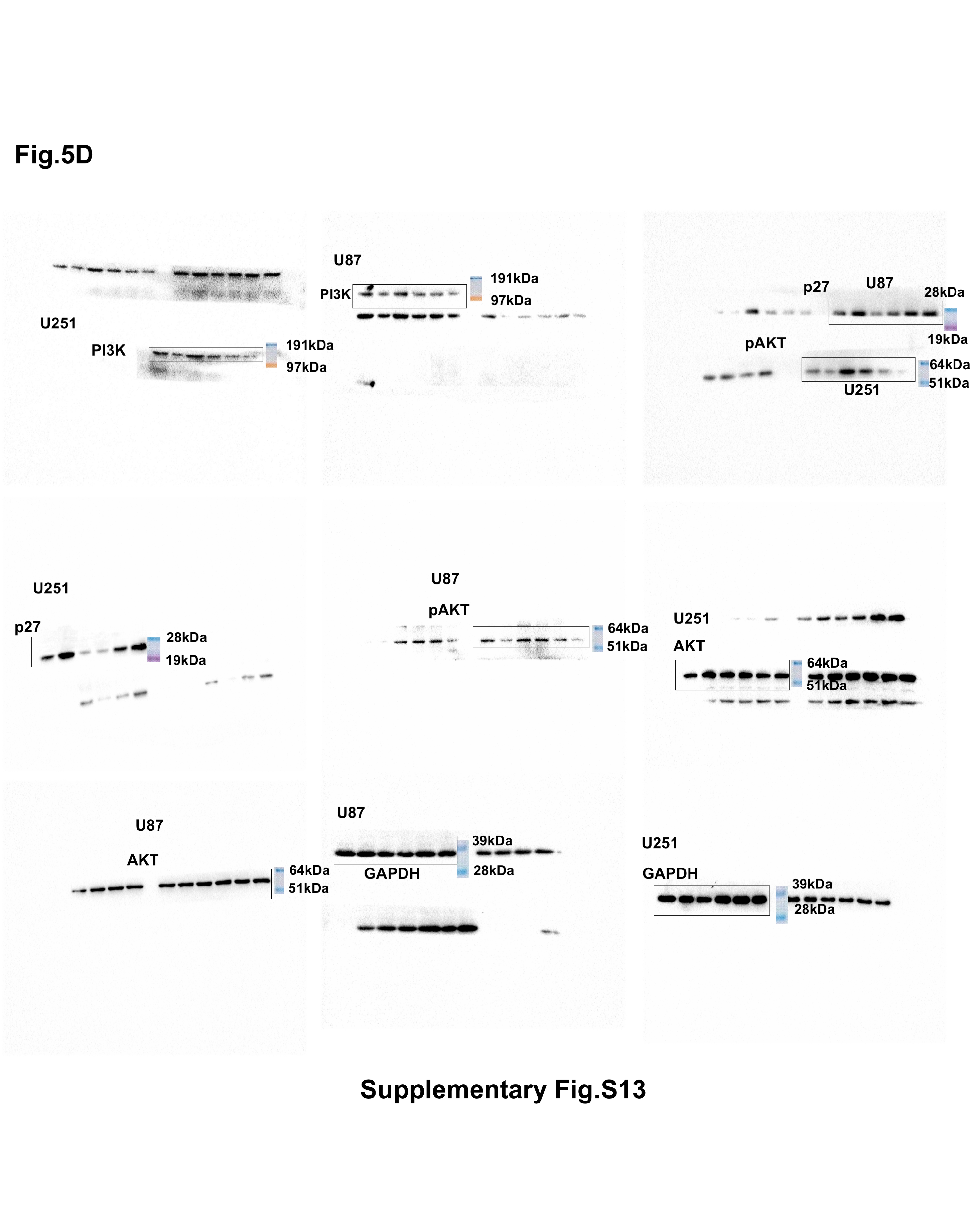

Supplement: Supplementary file 13 [file CAM4-7-4729-s013.tif]
