# Supplementary material for: Matrine induces senescence of human glioblastoma cells through suppression of the IGF1/PI3K/AKT/p27 signaling pathway
Source: Cancer Med. 2018 Aug 5;7(9):4729–43. doi: 10.1002/cam4.1720 (PMC6143938; doi:10.1002/cam4.1720)
Supplement: Supplementary file 14 [file CAM4-7-4729-s014.docx]

**Figure legends**

**Figure 1. Matrine inhibits proliferation and induces cell cycle arrest in GBM cells.** (**A**) Growth curves generated with cell viability results determined from the CCK-8 assay for normal human astrocytes (NHA), U251, U87, and P3 cells treated with different concentrations of matrine for 24, 48, and 72 h. Data points represent the percentage (%; OD450 untreated/OD450 treated) relative to untreated cells at that time point. (**B**) IC_50_ values for matrine in three glioma cell lines and NHA. (**C**) U251, U87, and P3 cells were treated with 0.2 mM matrine for 72 h. The cells were stained with Apollo 747 (red, representative of EdU) and the nuclear specific dye DAPI (blue). Scale bars = 20 μm. (**D**) Graphic representation of the percentage of EdU positive U251, U87, and P3 cells treated with different concentrations of matrine for 72 h. Statistical analyses were performed using Permutation Test. *P-*value came from the comparison between treated group and control group. All data are representative of 3 independent experiments.

**Figure 2. Matrine induces cellular senescence in GBM cells.** (**A**) Flow cytometric analysis of apoptosis in cells treated with 0.2mM matrine for 72 h as determined by annexin V- and/or FITC and propidium iodide staining for DNA content. The percentages of annexin V- and/or FITC-positive cells are indicated. (**B**) Western blot analysis of cas-3, cleaved cas-3, PARP, cleaved PARP and β-tublin expression in protein lysates (20 µg) prepared from U251 cells treated with matrine (0.2 mM) for the indicated days. (**C**) U251 cells in different phases of the cell cycle based on flow cytometric analysis (propidium iodide staining) of cells treated with 0.2 mM matrine for 72 h. All data are expressed as the mean ± the SD of values from experiments performed in triplicate. (D) Immunofluorescence staining for γH2AX (red) used to detect DNA damage and abnormal chromatin accumulated in U251 cells, U87, and P3 cells treated with matrine (0.2 mM) for 72 h. Cell nuclei were counterstained with DAPI (blue). Scale bars = 20 μm. (**E**) Graphic representation of cell number and γH2AX-positive content U251, U87, and P3 cells treated with 0.2 mM matrine for 72 h. Statistical analyses were performed using Permutation Test. *P-*value came from the comparison between treated group and control group. (**F**) *In situ* SA-β-gal assay to detect senescent cells. U251, U87 and P3 cells were treated with 0.2 mM marine for 72 h. Cellular senescence was examined by SA-β-gal staining. Scale bars = 20 μm. (**G**) Graphic representation of the percentage of SA-β-gal positive cells determined in four random fields per sample. All data are expressed as the mean ± the SD of values from experiments performed in triplicate. Statistical analyses were performed using Permutation Test. *P-*value came from the comparison between treated group and control group.

**Figure 3. Matrine induces cellular senescence by upregulating p27.** (**A**) Western blot analysis for cell cycle associated proteins in lysates prepared from U251 and U87 cells treated with matrine for the indicated number of days. The immunoblots are representative of at least two independent experiments with GAPDH serving as a protein loading control. (**B**) Western blot analysis of p27, CDK4 and CDK6 expression in U251 and U87 cells transfected with p27 siRNA for 48 h. (**C**) *In situ* SA-β-gal assay to detect senescent cells. Cells were treated with 0.2 mM matrine or p27 siRNA for 72h and compared to controls. Scale bars = 20 μm. (**D**) Graphic representation of the percentage of SA-β-gal positive cells determined in four random fields per sample. All data are expressed as the mean ± the SD of values from experiments performed in triplicate. Statistical analyses were performed using Permutation Test. *P*-value came from the comparison between treated group and control group or between two treated groups.

**Figure 4. Matrine inhibits the PI3K/AKT/p27 signaling pathway.** (**A**) Western blot analysis for PI3K, AKT, pAKT, and GAPDH protein levels in protein lysates (20 µg) prepared from U251 and U87 cells treated with matrine for the number of days indicated. (**B**) Western blot analysis to detect levels of AKT, pAKT, P27 and GAPDH in lysates prepared from U251 and U87 cells treated with matrine (0.2 mM), AKT activator SC79 (5 μg/mL), or PI3K inhibitor LY294002 (1 mM) 72 h. (**C)** *In situ* SA-β-gal assay to detect senescent cells. Cells were treated with 0.2 mM matrine and AKT activator SC79 for 72 h. Scale bars = 20 μm. (**D**) Graphic representation of the percentage of SA-β-gal positive cells determined in four random fields per sample. All data are expressed as the mean ± SD of values from experiments performed in triplicate. Statistical analyses were performed using Permutation Test. *P*-value came from the comparison between treated group and control group or between two treated groups.

**Figure 5. Downregulation of IGF1 enhances matrine-induced cellular senescence in GBM cells.** (**A**) Cytokine array to detect secreted protein levels in controls and matrine-treated U251 for 72 h. (B) Fold expression levels of IGF1 as determined in the cytokine assay. Chemiluminescent signals quantified with ImageJ software. (**C**) Results from ELISA performed to detect levels of IGF1 in media collected from control and matrine-treated U251 and U87 cells. Statistical analyses were performed using Permutation Test. *P*-value came from the comparison between treated group and control group. (**D**) Western blot analysis of PI3K, pAKT, AKT, p27 and GAPDH expression in U251 and U87 cells treated with 0.2 mM matrine, exogenous IGF1 (200 ng/mL), or IGF1 antibody (1 mg/mL) for 72 h. (**E**) *In situ* SA-β-gal assay to detect senescent cells. Cells were treated with matrine, exogenous IGF1, or IGF1 antibody for 72 h. Scale bars = 20 μm. (**F**) Graphic representation of the percentage of SA-β-gal-positive cells as determined in four random fields per sample. All data are expressed as the mean ± the SD of values from experiments performed in triplicate. Statistical analyses were performed using analysis of variance in randomized blocks. *P*-value came from the comparison between treated group and control group or between two treated groups.

**Figure 6.** **Matrine inhibits growth and induces cellular senescence of U251 GBM cells *in vivo*.** (**A**) U251 cells expressing luciferase were orthotopically implanted into athymic nude mice. Tumor growth was monitored using the IVIS-200 imaging system for detection of bioluminescence. Bioluminescent signals were measured at days 7, 14, 21, and 28 after implantation. (**B**) Bioluminescence values plotted as a function of time in days to assess tumor growth (days 7, 14, 21, and 28). Statistical analyses were performed using Permutation Test. *P-*value came from the comparison between treated group and control group. (**C**) Overall survival as determined by Kaplan-Meier survival curves. A log-rank test was used to assess the statistical significance of the differences (*P* < 0.05). (**D**) P3 cells expressing luciferase were orthotopically implanted into athymic nude mice and tumor growth was monitored using the IVIS-200 imaging system for detection of bioluminescence. Bioluminescent signals were measured at days 7, 14, 21, and 28 after implantation. (**E**) Bioluminescence values plotted as a function of time in days to assess tumor growth (days 7, 14, 21 and 28). Statistical analyses were performed using Permutation Test. *P*-value came from the comparison between treated group and control group. (**F**) Overall survival as determined by Kaplan-Meier survival curves. A log-rank test was used to assess the statistical significance of the differences (*P* < 0.01). (**G**) Graphic representation of percentage of Ki67, H2AX, and p27 positive cells in control and matrine-treated U251 xenografts as determined with immunohistochemistry. Statistical analyses were performed using Permutation Test. *P*-value came from the comparison between treated group and control group. (**H**) Graphic representation of percentage of Ki67, H2AX, and p27 positive cells in control and matrine-treated P3 xenografts as determined with immunohistochemistry. All data are expressed as the mean ± the SD of values from experiments performed in triplicate. Statistical analyses were performed using Permutation Test. *P*-value came from the comparison between treated group and control group.

**Figure 7.** **Proposed model for mechanism of matrine activity on human GBM cells**. Matrine suppresses GBM cell growth and invasion by inhibiting PI3K/AKT/p27 signaling and inducing senescence. Decreased IGF1 in matrine-induced senescent cells enhances response to matrine.

**Supplementary Figure S1. Matrine does not inhibit proliferation or cause apoptosis of NHA cells.** (**A**) Cells were stained with Apollo 647 (red, representative of EdU) and the nuclear specific dye DAPI (blue). Scale bars = 20 μm. (**B**) Graphic representation of the percentage of EdU positive NHA cells treated with different concentrations of matrine for 72 h. Statistical analyses were performed using Permutation Test. *P*-value came from the comparison between treated group and control group. (**C**) Flow cytometric analysis of apoptosis in cells treated with 0.2 mM matrine for 72 h as determined by annexin V- and/or FITC and propidium iodide staining for DNA content. The percentages of annexin V- and/or FITC-positive cells are indicated. All data are representative of 3 independent experiments.

**Supplementary Figure S2. Matrine does not induce apoptosis in GBM cell lines *in vitro*.** (**A**) Flow cytometric analysis of apoptosis in cells treated with 0.2mM matrine for 72 h as determined by annexin V- and/or FITC and propidium iodide staining for DNA content. The percentages of annexin V- and/or FITC-positive cells are indicated. (**B**) Western blot analysis of cas-3, cleaved cas-3, PARP, cleaved PARP and β-tubulin expression in protein lysates (20 µg) prepared from U87 and P3 cells treated with matrine (0.2 mM) for 72 h. (**C**) Immunofluorescence (FITC) TUNEL assay to detect apoptotic cells *in situ*. Nuclei are labeled with DAPI. Scale bars = 20 μm. All data are representative of 3 independent experiments.

**Supplementary Figure S3.** (**A**) Percentage of matrine treated (0.2 mM for 72 h) U87 and P3 cells in different phases of the cell cycle based on flow cytometric analysis (propidium iodide staining). (**B**) *In situ* SA-β-gal assay to detect senescent cells. NHA cells were treated with 0.2 mM marine for 72 h. Cellular senescence was examined by SA-β-gal staining. Scale bars = 20 μm. (**C**) Graphic representation of the percentage of SA-β-gal positive cells determined in four random fields per sample. All data are expressed as the mean ± the SD of values from experiments performed in triplicate. Statistical analyses were performed using Permutation Test. *P*-value came from the comparison between treated group and control group.

**Supplementary Figure S4.** **Matrine interaction molecular network.** (**A & B**) The predicted molecular network of matrine obtained from BATMAN.

**Supplementary Figure S5.** (**A**) Flow cytometric analysis of apoptosis in LN18 and LN229 cells treated with 0.2 mM matrine for 72 h as determined by annexin V- and/or FITC and propidium iodide staining for DNA content. The percentages of annexin V- and/or FITC-positive cells are indicated. (**B**) *In situ* SA-β-gal assay to detect senescent cells. LN18 and LN229 cells were treated with 0.2 mM marine for 72 h. Cellular senescence was examined by SA-β-gal staining (scale bars 20 μm). (**C**) Graphic representation of the percentage of SA-β-gal positive cells determined in four random fields per sample. All data are expressed as the mean ± the SD of values from experiments performed in triplicate. Statistical analyses were performed using Permutation Test. *P*-value came from the comparison between treated group and control group.

**Supplementary Figure S6.** Images of immunohistochemical staining for Ki67, γH2AX and p27 in tumors from each group as indicated (scale bars 20 μm).

**Supplementary Figure S7. Gating strategy used in flow cytometry analysis to detect cell apoptosis.** U251, NHA, U87, P3, LN18, and LN229 populations were gated on a forward scatter (FSC)/side scatter (SSC) plot. Early and late apoptotic cells were further gated to determine cell apoptosis (Fig. 2A, Supplementary Fig. S1C, S2A and S5A). All data are representative of 3 independent experiments.

**Supplementary Figure S8. Gating strategy used in flow cytometry analysis to determine cell cycle parameters.** U251, U87, and P3 populations were gated on a forward scatter (FSC)/side scatter (SSC) plot. Live cells were then further gated on FL2-H/FL2-A. Cells were further gated to determine cell cycle. All data are representative of 3 independent experiments.

**Supplementary Figure S9-S13.** Whole western blot gel scans. Bands were quantitated and results presented as a ratio using GAPDH as the loading control.
